# EPI-CKD Brazil: point-of-care screening for chronic kidney disease during World Kidney Day

**DOI:** 10.1590/2175-8239-JBN-2025-0298en

**Published:** 2026-04-27

**Authors:** Viviane Calice-Silva, Patrícia Abreu, Maria Eliete Pinheiros, Ana Katarina de Cerqueira Delgado Lopes, Karla Cristina Petruccelli, Ana Wanda Barreto Marinho, Ana Flávia Moura, Felipe Costa Neves, Cláudia Maria Costa de Oliveira, Silvana Daher Costa, Luiz Roberto de Sousa Ulisses, Fábio Humberto Ribeiro Paes Ferraz, Ramiele Aparecida Cruz Souza, Rachel Gatti Armani, Ciro Bruno Costa, Marcelo Garcia Tavares, Dyego José de Araújo Brito, Erika Cristina Ribeiro de Lima Carneiro, Luiz Gonzaga, Jenaine Oliveira Paixão, Helady Sanders-Pinheiro, Luis Claudio Santos Pinto, Lucas Acatauassu Nunes, Pablo Rodrigues Costa Alves, Amanda Maíra Damasceno Silva, René Scalet dos Santos, Suzana Ricardo Greffin, Gisele Vajgel, João Marcelo Medeiros de Andrade, Ginivaldo Victor Ribeiro do Nascimento, Igor Denizarde Bacelar Marques, Maria Izabel de Holanda, Pedro Tulio Monteiro de Castro Abreu Rocha, Kalyanne Cabral de Paula do O, Lelyanne Rodrigues Pereira Torauato, Cristina Karohl, Juliana Alves Manhaes de Andrade, Ariane Karen de Sousa, Denise Rodrigues Simão, Ana Carolina Nakamura Tome, Daniela Ponce, Fernanda Salomão Gorayeb-Polacchini, Maria Almerinda Vieira Fernandes Ribeiro Alves, Rubens Escobar Pires Lodi, Cinthia Montenegro, José A. Moura-Neto

**Affiliations:** 1 Sociedade Brasileira de Nefrologia, São Paulo, SP, Brazil.; 2 Universidade da Região de Joinville, Escola de Medicina, Joinville, SC, Brazil.; 3 Fundação Pró-Rim, Departamento de Pesquisa Científica, Joinville, SC, Brazil.; 4 Universidade Federal de São Paulo, São Paulo, SP, Brazil.; 5 Universidade Federal de Alagoas, Faculdade de Medicina, Maceió, AL, Brazil.; 6 Universidade Federal do Amazonas, Manaus, AM, Brazil.; 7 Escola Bahiana de Medicina e Saúde Pública, Salvador, BA, Brazil.; 8 Universidade Federal do Ceará, Hospital Geral de Fortaleza, Fortaleza, CE, Brazil.; 9 Nefroclínicas, Brasília, DF, Brazil.; 10 Universidade do Distrito Federal, Escola Superior de Ciências da Saúde, Brasília, DF, Brazil.; 11 Hospital Universitário Cassiano Antônio de Moraes, Vitória, ES, Brazil.; 12 Santa Casa de Misericórdia de Vitória, Escola Superior de Ciências, Vitória, ES, Brazil.; 13 Universidade Federal do Maranhão, Hospital Universitário, São Luís, MA, Brazil.; 14 Universidade Federal de Minas Gerais, Hospital das Clínicas, Belo Horizonte, MG, Brazil.; 15 Universidade Federal de Minas Gerais, Faculdade de Medicina, Belo Horizonte, MG, Brazil.; 16 Universidade Federal de Juiz de Fora, Faculdade de Medicina, Departamento de Clínica Médica, Juiz de Fora, MG, Brazil.; 17 Universidade Federal do Pará, Belém, PA, Brazil.; 18 Universidade Federal da Paraíba, Departamento de Medicina Interna, João Pessoa, PB, Brazil.; 19 Faculdades Pequeno Príncipe, Curitiba, PR, Brazil.; 20 Fundação Pró-Renal, Curitiba, PR, Brazil.; 21 Universidade Federal de Pernambuco, Recife, PE, Brazil.; 22 Instituto de Medicina Integral Prof. Fernando Figueira, Recife, PE, Brazil.; 23 Universidade Federal do Piauí, Teresina, PI, Brazil.; 24 Universidade Federal do Piauí, Hospital Universitário, Teresina, PI, Brazil.; 25 Hospital Federal de Bonsucesso, Rio de Janeiro, RJ, Brazil.; 26 Universidade Federal do Rio de Janeiro, Rio de Janeiro, RJ, Brazil.; 27 Rede Américas, Rio de Janeiro, RJ, Brazil.; 28 Hospital São Lucas Copacabana, Rio de Janeiro, RJ, Brazil.; 29 Universidade Federal do Rio Grande do Norte, Hospital Universitário Onofre Lopes, Natal, RN, Brazil.; 30 Universidade Federal de Ciências da Saúde de Porto Alegre, Porto Alegre, RS, Brazil.; 31 Santa Casa de Misericórdia de Porto Alegre, Serviço de Nefrologia, Porto Alegre, RS, Brazil.; 32 Universidade Federal do Rio Grande do Sul, Porto Alegre, RS, Brazil.; 33 Associação Renal Vida, Blumenau, SC, Brazil.; 34 Faculdade de Medicina de São José do Rio Preto, Hospital de Base, São José do Rio Preto, SP, Brazil.; 35 Universidade Estadual Paulista, Faculdade de Medicina de Botucatu, Botucatu, SP, Brazil.; 36 Universidade Estadual de Campinas, Faculdade de Ciências Médicas, Campinas, SP, Brazil.; 37 Nova Biomedical, Waltham, MA, USA.; 38 AstraZeneca Brasil, Cotia, SP, Brazil.

**Keywords:** Chronic kidney disease, Prevalence, Risk Factors, Mass Screening, Glomerular Filtration Rate

## Abstract

**Introduction:**

Chronic kidney disease (CKD) is a public health issue, with an estimated prevalence of 10% in Brazil, possibly underestimated due to regional inequalities and diagnostic limitations. The EPI-CKD Brazil study aimed to estimate the prevalence of estimated glomerular filtration rate (eGFR) < 60 mL/min/1.73 m^2^ in adults with risk factor for CKD, using point-of-care creatinine testing (POCTcr) during World Kidney Day 2025, and to assess regional variations and associated predictors.

**Methods:**

We conducted a multicenter cross-sectional study across 20 state chapters of the Brazilian Society of Nephrology, including individuals ≥ 18 years with at least one CKD risk factor (age > 60 years, hypertension [HTN], diabetes mellitus [DM], cardiovascular disease, obesity, chronic use of nonsteroidal anti-inflammatory drugs, history of acute kidney injury, bilateral kidney stones, or family history of CKD). Renal function was assessed using a rapid creatinine test (NovaMaxPro^®^–eGFR CKD-EPI 2021). Reduced eGFR was defined as < 60 mL/min/1.73 m^2^.

**Results:**

We analyzed 8,374 participants (66.9% women; median age, 58 years; BMI, 28.3 kg/m^2^; 46.2% mixed race). The frequency of reduced eGFR was 40.2% (n = 3,370), of whom 36% were in stages G3b–G5. Only 35.2% were aware of CKD risk factors. Significant regional differences were observed (ranging from 27.9% [Bahia] to 55.2% [Paraná]; p < 0.001). Independent predictors included age (OR = 1.032; 95%CI: 1.028–1.035), HTN (OR = 1.27; 95%CI: 1.15–1.40), and indigenous ethnicity as a protective factor (OR = 0.40; 95%CI: 0.20–0.89; p < 0.05). Risk increased by 23% for each additional risk factor.

**Conclusion:**

A high frequency of reduced eGFR and low awareness of CKD risk factors were observed. The study demonstrates the feasibility of POCTcr in screening strategies and reinforces the need for public policies to expand early diagnosis and strengthen primary care.

## Introduction

Chronic kidney disease (CKD) is a silent and progressive condition with an estimated global prevalence of approximately 10%^1,2^. In Brazil, observational studies indicate a prevalence ranging from 8.9% to 11% in the general population^3,4,5^. Considering the country’s vast geographic area, socioeconomic disparities, and unequal access to healthcare services and diagnostic testing, the true prevalence is likely underestimated^2^. Understanding the epidemiology of CKD is essential for strategic planning and public policy aimed at prevention and early detection^2,6^. These strategies are particularly relevant among high-risk populations, including individuals with hypertension, diabetes mellitus, cardiovascular disease (CVD), obesity, age > 60 years, and those with a family history of kidney disease, among other risk factors^7^.

Recent data from the 2024 Brazilian Dialysis Survey demonstrate a growing demand for kidney replacement therapy (KRT): as of July 2024, approximately 172,585 patients were undergoing chronic dialysis (~812 per million population), with 52,944 new patients initiating dialysis in that year; notably, only ~5.6% were treated with peritoneal dialysis^8^. In parallel, the IMPACT CKD model projects that between 2023 and 2032, Brazil will experience a substantial increase in CKD—particularly in stages 3–5 (~6.9% growth)—resulting in a larger number of individuals requiring KRT, along with marked increases in morbidity, mortality, and economic burden^9^. Taken together, these findings underscore the immense clinical, human, and economic burden of CKD on the Brazilian healthcare system. The rising incidence and prevalence of advanced CKD exceed the country’s treatment capacity, while many early-stage cases remain undiagnosed. This mismatch highlights the urgent need to strengthen screening in primary care, expand access to diagnostic testing (including laboratory and point-of-care tests), and implement prevention strategies targeting risk factors such as diabetes, hypertension, and obesity. Proactive public policies and investments in early detection could mitigate progression to kidney failure, reduce costs, and alleviate the growing strain on dialysis infrastructure nationwide.

Currently, CKD diagnosis is based on the estimated glomerular filtration rate (eGFR), calculated by the CKD-EPI (2021) equation using serum creatinine, and on the detection of albuminuria through the urine albumin-to-creatinine ratio (uACR) in a spot urine sample. CKD is defined by the presence of eGFR < 60 mL/min/1.73 m^2^ and/or a uACR > 30 mg/g, persisting for more than three months, with or without structural renal abnormalities. Classification follows the system proposed by Kidney Disease: Improving Global Outcomes (KDIGO), originally published in 2012 and updated in 2024^7^, combining eGFR and uACR categories to stage the disease, stratify the risk of progression, and guide referral to nephrology care. As CKD progresses to more advanced stages, complications may arise, potentially culminating in kidney failure and the need for kidney replacement therapy (KRT)^7^.

However, one of the major challenges in early detection is the difficulty of implementing large-scale screening strategies, as most diagnostic tests require minimal laboratory infrastructure for creatinine and uACR testing. In this context, bedside or community-based screening tools—particularly in primary healthcare settings—may help overcome the absence of systematic screening and promote timely referral^10,11^. World Kidney Day (WKD) is the largest global campaign dedicated to kidney health awareness^12^. In Brazil, the Brazilian Society of Nephrology (BSN) has coordinated national outreach activities for over two decades, involving its state chapters in health education initiatives, risk-factor screening, and community engagement. In 2025, the campaign reached all 20 active BSN state chapters, aiming to raise public awareness of CKD and integrate scientific and public health initiatives, and, for the first time, incorporated nationwide CKD screening using rapid creatinine testing.

Point-of-care diagnostic tests (POCT) have emerged as feasible tools to assess kidney function outside hospital settings^13^. Portable devices capable of measuring capillary creatinine levels and estimating eGFR in real time enable screening in public health campaigns, primary care clinics, emergency services, and even in remote areas^13^. Despite variability in accuracy and precision across devices, international experience indicates that point-of-care creatinine testing (POCTcr) expands screening coverage and contributes to reducing delays in CKD diagnosis^14,15,16,17^.

Therefore, the EPI-CKD Brazil study aimed to estimate the prevalence of eGFR < 60 mL/min/1.73 m^2^ among adults with CKD risk factors through screening of a convenience sample using POCTcr across all regions of the country. The screening was conducted during the 2025 World Kidney Day campaign, coordinated by the BSN and its state chapters. Secondary objectives included assessing regional variations in the prevalence of eGFR < 60 mL/min/1.73 m^2^, identifying major predictors associated with reduced eGFR, and investigating population awareness of CKD risk factors.

## Methods

### Study Design

This is a multicenter, cross-sectional study using a convenience sample, conducted during the 2025 WKD campaign in Brazil. Due to its cross-sectional design and the use of a single eGFR measurement obtained via POCTcr, the study does not allow for a definitive diagnosis of CKD, but it does enable the identification of reduced eGFR values potentially compatible with the disease. Therefore, participants could only be classified as having reduced kidney function or as having a positive screening for CKD, rather than as CKD patients.

### Study Sites and Participants

The study was conducted as part of the 2025 WKD campaign actions in the 20 state chapters of the BSN, covering 20 of the 27 federative units of Brazil. The participant inclusion period ran from March 11 to April 30, 2025. As this study was carried out within a kidney disease awareness campaign, individuals were approached and invited to participate in public places where campaign activities were held. This approach may have introduced selection bias, given the different settings in which the campaign was conducted (e.g., shopping centers, public squares, among others). The recruitment cities were defined by the BSN state chapters board that registered to participate in the study.

Adult individuals (≥ 18 years) with at least one risk factor for CKD were included. The risk factors considered were (1) age > 60 years; (2) hypertension (HTN); (3) diabetes mellitus (DM); (4) cardiovascular disease (CVD); (5) obesity (BMI ≥ 30 kg/m^2^); (6) history of acute kidney injury (AKI); (7) history of bilateral renal lithiasis; (8) history of recurrent urinary tract infection; (9) chronic use of nonsteroidal anti-inflammatory drugs (NSAIDs); and (10) family history of CKD. The following were excluded: protein intake > 25 g within four hours prior to the creatinine test; continuous use of protein- and/or creatine-based nutritional supplements; prior diagnosis of CKD of any etiology; current KRT (hemodialysis, hemodiafiltration, peritoneal dialysis, or kidney transplantation); and participants with incomplete information in the data collection platform. Only individuals who agreed to participate and signed the informed consent form (ICF) were included. The study was approved by the Research Ethics Committee of the Hans Dieter Schmidt Hospital (CAAE: 86100725.1.1001.5363).

### Procedures

After signing the ICF, each participant answered a standardized questionnaire for the collection of demographic data, risk factors, and comorbidities, in addition to a question about their knowledge of CKD risk factors (Question: “Do you know the main risk factors for developing chronic kidney disease?” ( ) Yes ( ) No). Next, POCTcr was performed via finger prick to obtain a single capillary blood sample using the Nova MaxPro^®^Creat/eGFR creatinine meter (Nova Biomedical, Waltham, MA, USA), a portable analyzer with a single-use miniaturized biosensor designed for creatinine measurement in whole blood. Individuals with eGFR < 60 mL/min/1.73 m^2^ were referred for subsequent diagnostic confirmation and specialist evaluation, according to the referral pathway established by each participating BSN state chapter.

Throughout this article, we refer to these individuals as having a “positive screening for CKD” to facilitate data interpretation. This designation was adopted because the methodology used does not meet the laboratory diagnostic criteria for CKD, which require evaluation of eGFR and/or urine albumin-to-creatinine ratio (uACR) in a spot urine sample on at least two occasions with a 90-day interval between them. Therefore, given the crosssectional nature of the study, CKD cannot be formally diagnosed in this population.

### Creatinine Measurement by Rapid Test

The Nova MaxPro^®^Creat/eGFR creatinine meter (Nova Biomedical, Waltham, MA, USA) allows for simple, rapid, and precise assessments of kidney function using a capillary blood sample obtained by finger pulp puncture. In previous studies, it showed a sensitivity of 98.9% and specificity of 85.3% for the detection of kidney disease when compared with the standard laboratory method. The device also performs GFR estimation using the CKD-EPI 2021 equation^16^.

### Collected Variables

Demographic, clinical, and CKD knowledge-related variables were collected and stored in a de-identified manner by the principal investigator on an electronic platform (REDCap), ensuring participant privacy. The demographic and anthropometric data collected were age, sex, self-reported race/ethnicity (white, black, brown [miscegenation between whites and blacks], indigenous, and Asian), body weight (kg), height (m), body mass index (BMI, kg/m^2^), physical activity, and smoking.

Comorbidities were defined as: prior diagnosis of HTN (systemic hypertension): systolic blood pressure ≥ 140 mmHg or diastolic blood pressure ≥ 90 mmHg, or reported use of antihypertensive medication; prior diagnosis of DM (diabetes mellitus): reported diagnosis or use of hypoglycemic medications; CVD (cardiovascular disease): history of coronary revascularization, myocardial infarction, stroke (CVA), and/or heart failure; obesity: BMI ≥ 30 kg/m^2^; reported chronic use of NSAIDs: weekly use; history of bilateral kidney lithiasis: documented by imaging examination; recurrent urinary tract infection: compatible symptoms associated with antibiotic treatment > 3 times/year; prior history of AKI (acute kidney injury): temporary worsening of kidney function; family history of CKD: first- or second-degree relatives undergoing conservative treatment for CKD or KRT (kidney replacement therapy).

### Statistical Analyses

Descriptive analyses were performed for numerical variables and presented according to their distribution. Categorical variables were presented as absolute and relative frequencies (n, %). Independentsamples chi-square tests and the Kruskal–Wallis test were used for the comparative analysis of prevalence across BSN state chapters and of the characteristics of individuals with and without positive screening for CKD (eGFR < 60 mL/min/1.73 m^2^). Logistic regression was utilized to identify factors associated with CKD. The criterion adopted for a variable’s inclusion in the logistic regression was a p-value of < 0.1 in the univariate analysis. The logistic regression model was not adjusted for clustering by BSN state chapter. The presence of collinearity among the independent variables was assessed using collinearity diagnostics via linear regression analysis, examining variance inflation factor (VIF) and tolerance values. VIF values > 5 and tolerance < 0.2 were considered indicative of significant collinearity. We also analyzed the additional risk of positive screening for CKD according to the number of risk factors, considering the most prevalent ones in the studied group (HTN, DM, age > 60 years, obesity and CVD). Data were analyzed using SPSS software, version 30.0 (IBM Corp., Armonk, NY, USA).

## Results

**Figure 1:**
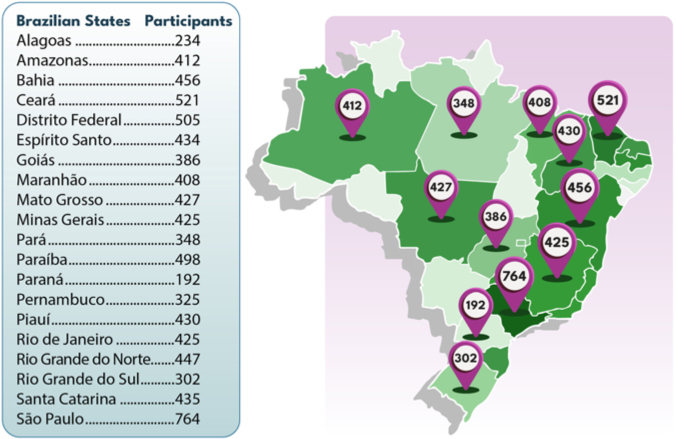
Distribution of participants in the study according to the BSN state chapters and the country’s federative units.

During the study period, 8,428 individuals were evaluated in 40 cities across 20 federative units (Figure 1). Of these, 8,374 participants had complete data and were included in the analyses. The sample was composed predominantly of women (66.9%), with a median age of 58 years and a median BMI of 28.3 kg/m^2^. Most participants self-declared as brown (*pardos*; 46.2%) (Table 1).

**Table 1:** Demographic, clinical, and creatinine measurement data of the population included in the study

Variable	N = 8374
Age [years, median (1^st^–3^rd^ quartile; min–max)]	58 (37–79) (18–103)
Female (n, %)	5574 (66.9)
Ethnicity	
White (n, %)	3078 (37.4)
Black (n, %)	1294 (15.7)
Brown (n, %)	3800 (46.2)
Indigenous (n, %)	37 (0.4)
Asian (n, %)	24 (0.3)
Active smoker (n, %)	1022 (12.3)
Sedentary (n, %)	4822 (58.2)
Elderly (> 60 years) (n, %)	3994 (47.7)
HTN (n, %)	4484 (54.1)
DM (n, %)	2358 (28.7)
CVD (n, %)	1200 (14.7)
Obesity (n, %)	2598 (31.8)
AKI (n, %)	80 (1.2)
Bilateral kidney stones (n, %)	285 (4.4)
Chronic use of NSAIDs (n, %)	312 (4.8)
Family history of CKD (n, %)	417 (6.4)
DM + HTN (n, %)	1685 (20.1)
DM + HTN + CVD (n, %)	418 (5.0)
DM + HTN + CVD + Obesity (n, %)	127 (1.5)
BMI [kg/m^2^, median (1^st^–3^rd^ quartile; min–max)]	28.3 (21.3–35.3) (13–64)
Creatinine [mg/dL, median (1^st^–3^rd^ quartile; min–max)]	1.1 (0.65–1.55) < 0.25 – > 7.0
eGFR [mL/min/1.73 m^2^, median (1^st^–3^rd^ quartile; min–max)]	65 (34 – > 90) (< 15 – > 90)
eGFR ranges (n, %)	
> 90 (n, %)	1441 (17.2)
60–89 (n, %)	3541 (42.3)
45–59 (n, %)	2180 (26.1)
30–44 (n, %)	968 (11.6)
15–29 (n, %)	185 (2.2)
< 15 (n, %)	50 (0.6)

Abbreviations – HTN: Hypertension; DM: Diabetes mellitus; CVD: Cardiovascular disease; AKI: Acute kidney injury; NSAIDs: Nonsteroidal anti-inflammatory drugs; BMI: Body mass index; eGFR: Estimated glomerular filtration rate; CKD: Chronic kidney disease. IQR: Interquartile range.

**Figure 2:**
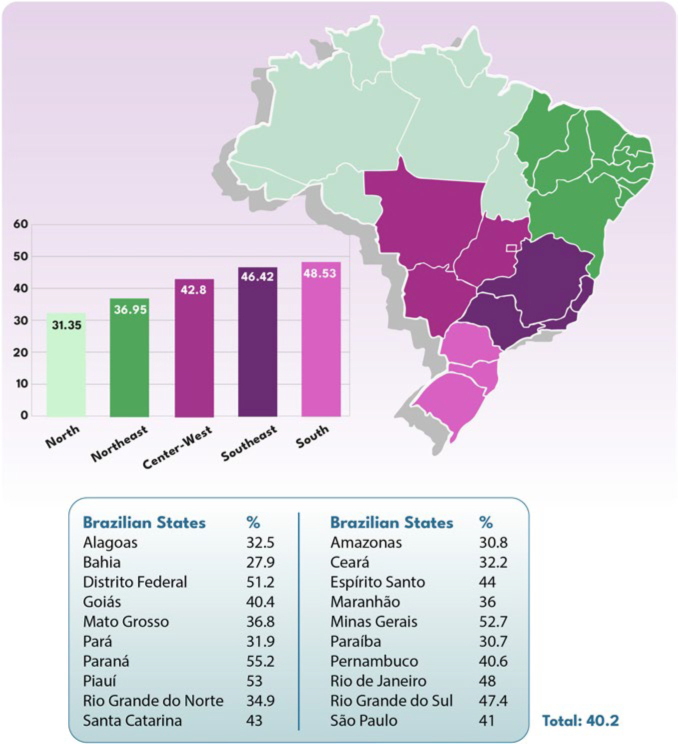
Distribution of study participants with eGFR < 60 mL/min/1.73 m^2^, according to A) BSN state chapters and B) the country’s federative units.

**Figure 3:**
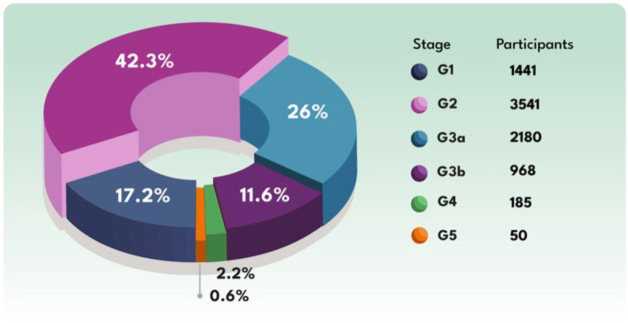
CKD stages in the studied population if the alteration in eGFR was confirmed.

The global frequency of reduced kidney function (eGFR < 60 mL/min/1.73 m^2^) and, consequently, positive screening for CKD, was 40.2% (n = 3,370). If CKD was confirmed, 36% of these individuals would be in stages G3b to G5 (Table 1; Figure 2A and B; and Figure 3). The comparison of the characteristics of the populations with and without reduced eGFR is presented in Table 2.

Abbreviation – G1 = ≥ 90 mL/min/1.73 m^2^; G2 = 60–89 mL/min/1.73 m^2^; G3a = 45–59 mL/min/1.73 m^2^; G3b = 30–44 mL/min/1.73 m^2^; G4 = 15–29 mL/min/1.73 m^2^; and G5 < 15 mL/min/1.73 m^2^.

**Table 2:** Comparison of the main characteristics of the population with and without reduced kidney function detected by the applied method

Variable	All N = 8374	eGFR ≥ 60 N = 5004	eGFR < 60 N = 3370	P-value^*^
Age [years, median (1^st^–3^rd^ quartile; min–max)]	58 (37–79) (18–103)	55 (22) (18–96)	62 (19) (18–103)	< 0.001
Female (n, %)	5574 (66.9)	3339 (67.2)	2235 (66.4)	0.43
Ethnicity				< 0.001
White (n, %)	3078 (37.4)	1755 (35.7)	1323 (39.8)	
Black (n, %)	1294 (15.7)	752 (15.3)	542 (16.3)	
Brown (n, %)	3800 (46.2)	2363 (48.1)	1437 (43.2)	
Indigenous (n, %)	37 (0.4)	28 (0.6)	9 (0.3)	
Asian (n, %)	24 (0.3)	12 (0.2)	12 (0.4)	
Active smoker (n, %)	1022 (12.3)	611 (12.3)	411 (12.2)	0.91
Sedentary (n, %)	4822 (58.2)	2928 (59.3)	1894 (56.5)	0.012
Elderly (> 60 years) (n, %)	3994 (47.7)	2017 (40.4)	1977 (58.4)	< 0.001
HTN (n, %)	4484 (54.1)	2432 (49.3)	2052 (61.1)	< 0.001
DM (n, %)	2358 (28.7)	1374 (28.1)	984 (29.5)	0.16
CVD (n, %)	1200 (14.7)	636 (13.0)	564 (17.0)	< 0.001
Obesity (n, %)	2598 (31.8)	1595 (32.7)	1003 (30.5)	0.03
Knowledge of risk factors (n, %)	2948 (35.2)	1851 (37.0)	1172 (34.8)	0.05
BMI [kg/m^2^, median (1^st^–3^rd^ quartile; min–max)]	28.3 (21.3–35.3) (13–64)	28.5 (21.5–35.5) (13–64)	28 (21–35) (15–56)	0.006
DM + HTN (n, %)	80 (1.2)	914 (18.3)	771 (22.8)	< 0.001
DM + HTN + CVD (n, %)	285 (4.4)	249 (4.4)	169 (5.8)	0.004
DM + HTN + CVD + Obesity (n, %)	312 (4.8)	72 (1.4%)	55 (1.6)	0.5
Creatinine [mg/dL, median (1^st^–3^rd^ quartile; min–max)]	1.1 (0.65–1.55) < 0.25 – > 7.0	0.94 (0.26) (< 0.25–1.63)	1.4 (0.4) (0.9 – > 7.0)	< 0.001
eGFR [mL/min/1,73 m^2^, median (1^st^–3^rd^ quartile; min–max)]	65 (34 – > 90) (< 15 – > 90)	78 (56 – > 90) (60 – > 90)	48 (35–59.5) (< 15 – 59.5)	< 0.001

Abbreviations – HTN: Hypertension; DM: Diabetes mellitus; CVD: Cardiovascular disease; BMI: Body mass index; eGFR: Estimated glomerular filtration rate. IQR: Interquartile range.

*P values refer to the comparison tests between CKD and non-CKD groups: qualitative variables were analyzed using independent samples chi-square tests, and quantitative variables using the Kruskal-Wallis independent samples test.

**Figure 4:**
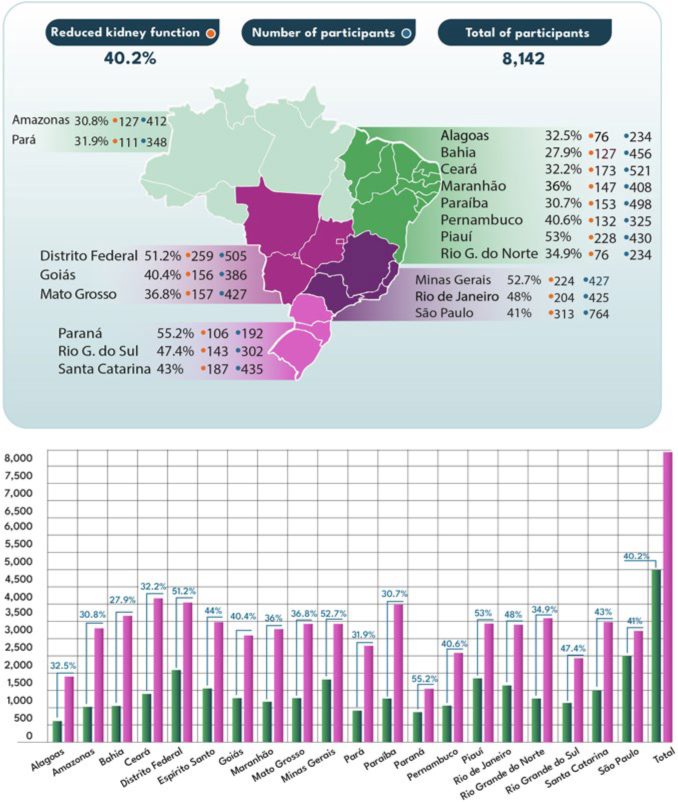
Prevalence of reduced kidney function (eGFR < 60 mL/min/1.73 m^2^) detected by creatinine POCT according to BSN state chapters.

The frequency of reduced kidney function was highest in Paraná (55.2%), Piauí (53%), the Federal District (51.3%), and Minas Gerais (52.7%), and lowest in Bahia (27.9%), Amazonas (30.8%), and Paraíba (30.7%), with a statistically significant difference between the state chapters (p < 0.001) (Figure 4). The general characteristics of the studied population according to each BSN state chapter varied considerably and are shown in Table 3.

**Table 3:** Comparison of participant characteristics among the bsn state chapters

Variable	All N = 8374	AL	AM	BA	CE	DE	ES	GO	MA	MT	MG	PA	PB	PR	PE	PI	RJ	RN	RS	SC	SP	p-value
Age [years, median (1^st^-3^rd^ quartile; min-max)]	58 (37-79)	52 (28-76)	54 (33-55)	59 (38-80)	50 (29-71)	53 (27-79)	50 (29-71)	56 (33-78)	53 (33-73)	50 (28-72)	63 (46-80)	53 (35-70)	62 (44-80)	67 (54-80)	61 (43-78)	62 (44-80)	57 (36-78)	69 (56-82)	61 (43-79)	54 (28-80)	63 (45-81)	< 0.001
Female (n, %)	5574 (66.9)	170 (73.9)	259 (63.30)	337 (74.1)	360 (69.2)	323 (65.6)	296 (68.2)	242 (63.2)	294 (72.1)	291 (68.1)	295 (69.6)	233 (67.0)	341 (68.8)	120 (62.5)	246 (75.7)	280 (65.3)	307 (72.7)	283 (63.5)	185 (61.5)	279 (64.3)	433 (56.7)	< 0.001
Ethnicity																						< 0.001
White (η, %)	3078 (37.4)	47 (22.5)	52 (13.0)	64 (14.1)	165 (32.3)	147 (29.5)	144 (33.2)	119 (30.8)	64 (15.7)	113 (26.5)	172 (41.3)	72 (20.7)	147 (29.8)	132 (76.3)	97 (30.6)	70 (17.2)	213 (51.7)	194 (43.9)	218 (72.2)	321 (74.1)	527 (69.3)	
Black (η, %)	1294 (15.7)	42 (20.1)	30 (75)	203 (44.5)	39 (76)	76 (15.2)	70 (16.1)	37 (9.6)	106 (26.0)	75 (27.6)	100 (24.0)	34 (9.8)	78 (15.8)	10 (5.8)	55 (17.4)	76 (18.7)	82 (19.9)	52 (11.8)	39 (12.9)	33 (76)	57 (7.5)	
Brown (η, %)	3800 (46.2)	119 (56.9)	310 (773)	188 (41.2)	306 (59.9)	269 (53.9)	218 (50.2)	229 (59.3)	236 (57.8)	234 (54.9)	142 (34.1)	241 (69.3)	264 (53.5)	29 (16.8)	164 (51.7)	259 (63.8)	117 (28.4)	196 (44.3)	43 (14.2)	78 (18.0)	158 (20.8)	
Indigenous (η, %)	37 (0.4)	1 (0.51	9 (2.2)	0	1 (0.2)	5(1.01	1 (0.2)	1 (0.3)	2 (0.51	3 (0.71	2 (0.51	1 (0.3)	3 (0.6)	2 (1.2)	1 (0.3)	1 (0.2)	0	0	2 (0.71	1 (0.2)	1 (0.1)	
Asian (η, %)	24 (0.3)	0	0	1 (0.2)	0	2 (0.4)	1 (0.2)	0	0	1 (0.2)	0	0	1 (0.2)	0	0	0	0	0	0	0	18 (2.4)	
Active smoker (n, %)	1022 (12.3)	18 (79)	53 (13.1)	53 (11.7)	68 (13.1)	70 (13.8)	34 (78)	45 (11.7)	19 (4.7)	68 (15.9)	78 (18.5)	35 (10.1)	76 (15.3)	12 (6.6)	32 (9.9)	44 (10.6)	53 (12.6)	49 (11.0)	33 (10.9)	35 (8.1)	147 (19.2)	< 0.001
Sedentary (n, %)	4822 (58.2)	125 (56.1)	260 (64.0)	272 (59.8)	302 (58.1)	274 (54.3)	261 (60.4)	229 (59.3)	273 (67.2)	316 (74.4)	235 (56.4)	231 (66.4)	288 (58.4)	64 (35.4)	183 (577)	231 (51.7)	214 (50.8)	273 (61.1)	126 (41.9)	240 (55.4)	443 (58.2)	< 0.001
Elderly (> 60 years) (n, %)	3994 (47.7)	83 (35.5)	157 (38.1)	225 (49.3)	244 (46.8)	153 (30.3)	165 (38.0)	150 (38.9)	129 (31.6)	116 (27.2)	268 (63.1)	109 (31.3)	295 (59.2)	153 (79.7)	178 (54.8)	256 (59.5)	195 (45.9)	244 (54.6)	241 (79.8)	172 (39.5)	461 (60.3)	< 0.001
HTN (n, %)	4484 (54.1)	131 (67.2)	189 (46.6)	239 (52.4)	252 (48.5)	182 (36.1)	236 (54.5)	170 (44.9)	205 (50.4)	205 (48.0)	268 (63.2)	172 (49.4)	311 (62.7)	102 (54.8)	197 (60.8)	229 (54.3)	212 (50.1)	269 (60.6)	207 (68.5)	236 (54.4)	472 (61.9)	< 0.001
DM (n, %)	2358 (28.7)	57 (35.4)	100 (24.6)	155 (34.2)	140 (27.0)	84 (16.7)	115 (26.5)	76 (20.1)	103 (25.2)	86 (20.1)	157 (37.3)	90 (25.9)	163 (32.9)	56 (29.9)	108 (33.4)	102 (25.0)	106 (25.1)	163 (37.3)	99 (32.9)	110 (25.3)	288 (378)	< 0.001
CVD (n, %)	1200 (14.7)	34 (22.7)	57 (14.1)	63 (13.8)	75 (14.5)	57 (11.4)	28 (6.5)	44 (11.6)	73 (18.0)	56 (13.1)	81 (19.2)	32 (9.2)	89 (17.9)	35 (19.3)	48 (14.9)	59 (14.9)	39 (9.3)	87 (20.2)	58 (19.2)	53 (12.2)	145 (19.1)	< 0.001
Obesity (n, %)	2598 (31.8)	122 (52.1)	149 (37.0)	111 (24.7)	194 (39.4)	138 (27.3)	152 (35.2)	129 (33.4)	125 (31.1)	166 (39.1)	122 (31.4)	109 (31.3)	182 (36.6)	53 (33.5)	91 (28.3)	112 (28.8)	142 (33.6)	90 (21.7)	18 (6.0)	164 (37.7)	229 (30.0)	< 0.001

Notes - P values refer to the comparison tests between CKD and non-CKD groups: qualitative variables were analyzed using independent samples chi-square tests, and quantitative variables using the Kruskal-Wallis test.

**Figure 5:**
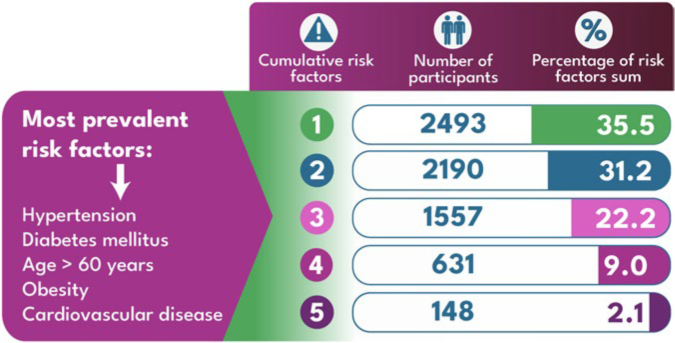
Distribution of the sum of the most prevalent CKD risk factors. Included: HTN, DM, age > 60 years, obesity, and CVD.

Only 35.2% of participants reported knowing the risk factors for CKD. Among the evaluated risk factors for CKD, the most frequent ones were HTN, age > 60 years, obesity, and DM (Table 2). The distribution of the sum of risk factors is shown in Table 2 and Figure 5. For each additional risk factor reported, the risk of positive screening for CKD increased by 23% (OR = 1.23; 95% CI: 1.20–1.30).

The main predictors for reduced eGFR were age (OR = 1.032; 95% CI: 1.028–1.035), arterial hypertension (OR = 1.27; 95% CI: 1.15–1.4), and indigenous ethnicity, which was found to be a protective factor against reduced eGFR (OR = 0.40; 95% CI: 0.20–0.89). These factors were all statistically significant (p < 0.05). Other logistic regression results are presented in Table 4. No significant collinearity was observed among the risk factors included in the model.

**Table 4:** Predictors of reduced glomerular filtration rate

Variable	Crude model		Model 1		Model 2	
	OR (95% CI)	p-value	OR (95% CI)	p-value	OR (95% CI)	p-value
Age	1.03 (1.02–1.04)	< 0.001	1.03 (1.02–1.04)	< 0.001	1.03 (1.02–1.04)	< 0.001
Sex	1.04 (0.96–1.14)	0.43				
Ethnicity						
White	Reference		Reference		Reference	
Black	0.96 (0.84–1.1)	0.5	1.07 (0.93–1.22)	0.34	1.03 (0.93–1.18)	0.7
Brown	0.81 (0.73–0.9)	< 0.001	0.94 (0.85–1.04)	0.2	0.94 (0.84–1.03)	0.15
Indigenous	0.43 (0.22–0.91)	< 0.001	0.45 (0.21–0.98)	0.05	0.4 (0.2–0.9)	0.03
Asian	1.33 (0.59–2.9)	0.5	1.07 (0.47–2.43)	0.8	1.2 (0.53–2.8)	0.6
Active smoker	0.99 (0.87–1.13)	0.9				
Sedentary	0.89 (0.82–0.97)	0.01	0.97 (0.88–1.06)	0.5	0.95 (0.87–1.05)	0.3
HTN	1.61 (1.47–1.76)	< 0.001			1.22 (1.1–1.35)	< 0.001
DM	1.07 (0.97–1.2)	0.16				
CVD	1.37 (1.21–1.55)	< 0.001			1.09 (0.96–1.25)	0.2
Obesity	0.9 (0.82–0.99)	0.03			1.09 (0.98–1.2)	0.1

Abbreviations – HTN = Systemic arterial hypertension; DM = Diabetes mellitus; CVD = Cardiovascular disease.

Model 1: Adjusted for sociodemographic variables that presented a p-value < 0.1 in the univariate analysis.

Model 2: Model 1 + the most prevalent comorbidities in the studied population.

## Discussion

The EPI-CKD Brazil study revealed a high prevalence of reduced kidney function (eGFR < 60 mL/min/1.73 m^2^) among individuals with risk factors. This was assessed using a rapid creatinine test and eGFR estimated by the CKD-EPI 2021 equation during the 2025 World Kidney Day (WKD) campaign actions across the 20 BSN state chapters. Due to the cross-sectional design of the study and the use of a single eGFR measurement via POCTcr, it is not possible to determine the prevalence of CKD, but rather to assess the risk of kidney disease and reduced eGFR at the time of evaluation^18,19,20^.

Surveys in China indicated a prevalence of 10.8% in 2009 and ~82 million adults with CKD in 2018–2019, suggesting temporal changes possibly associated with public policies encouraging early diagnosis^21^. In Latin America, data from the ISN Global Kidney Health Atlas indicate an average prevalence of ~10%, with wide variability among countries. In Brazil, observational studies indicate a prevalence in the general population of around 8.9% to 11%^2,3^, as well as an underutilization of uACR in the diagnosis of CKD in the country^4^.

It is estimated that among patients with CKD risk factors, the global prevalence ranges between 20% and 30%, depending on the specific risk profile and the region studied^22,23,24^. In a study by the International Society of Nephrology’s Kidney Disease Data Center (ISN-KDDC), that included 75,058 individuals from 12 low- and middle-income countries, the prevalence of CKD assessed using combined eGFR and uACR was 14.3% (95% CI: 14.0–14.5) in the general population and 36.1% (95% CI: 34.7–37.6) in high-risk populations (individuals with HTN, DM, or CVD)^6,25^. In that study, prevalences in high-risk individuals ranged from 19.4% in Georgia, Eastern Europe, to 49.3% in Bangladesh, South Asia^25^. Furthermore, data from the KEEP (Kidney Early Evaluation Program) conducted in the USA demonstrated that CKD is highly prevalent among individuals with known risk factors for the disease. Nearly 30% showed signs of early kidney damage (albuminuria), and 16% had reduced kidney function, of whom 14% had newly diagnosed CKD^26^. In Brazil, prevalence data among patients with CKD risk factors are still scarce.

For the first time in Brazil, adults with risk factors for CKD had access to large-scale screening via POCTcr. Although universal population screening is debatable in terms of cost-effectiveness, the combined use of eGFR and uACR is recommended for early detection in risk groups. This approach allows optimized CKD management with interventions capable of reducing disease progression^7^. However, economic disparities and variations in access to health services and diagnostic capabilities have a profound impact on the epidemiology, progression, and outcomes of kidney disease worldwide. These disparities are evident both in low- and middle-income countries (LMICs) and in high-income countries, even in those with universal healthcare systems, such as Brazil^7,11,27^.

Considering the CKD risk factors analyzed in the studied population, the majority of participants were elderly (> 60 years), hypertensive (HTN), diabetic (DM), and obese. Each added risk factor increased the risk of a positive screening for CKD by 23%. In Brazil, data from population surveys demonstrate the relevance of the main CKD risk factors in the elderly population. These data come from sources such as the Surveillance System of Risk and Protective Factors for Chronic Diseases by Telephone Survey (VIGITEL) and demographic surveys conducted by the Brazilian Institute of Geography and Statistics (IBGE)^28,29^. Currently, approximately 15% of the Brazilian population is aged 60 years or older, an age group in which the prevalence of HTN exceeds 50% and DM approaches 20%, according to VIGITEL estimates^28^. Additionally, approximately one-quarter of Brazilians in this age group have obesity, reflecting the impact of population aging associated with lifestyles that favor chronic diseases^28,29^. These findings reinforce the growing burden of cardiometabolic conditions and their role in determining the prevalence and progression of CKD in the country.

Early detection of CKD through POCT is key to identifying individuals at risk. These tests primarily aim to provide rapid assessment of kidney function (capillary creatinine with eGFR calculation performed on the device itself) and the detection of kidney injury through the assessment of albumin in the urine. In CKD screening and monitoring, POCTcr can be utilized to stratify patients when venous sampling or central laboratories are not readily available (e.g., outreach clinics, radiology departments, and emergency rooms), although accuracy and precision vary according to the equipment and clinical context^30,31,32^.

In our study, all participants with eGFR < 60 mL/min/1.73 m^2^ were referred for laboratory confirmation through serum creatinine testing. Those with results consistent with CKD were referred for medical evaluation according to CKD stage, as per the 2024 Clinical Protocols and Therapeutic Guidelines (PCDT)^33^ and the referral pathway organized by each BSN state chapter. However, these data were not captured in the present study, making it impossible to confirm the diagnosis of CKD based on our data. The Nova MaxPro^®^Creat/eGFR rapid test showed high sensitivity (98.9%) and specificity (85.3%), but these values do not rule out the possibility of false-positive results and indicate the need for subsequent confirmation by a gold-standard laboratory test^16^. Data from the literature on other point-of-care creatinine testing devices reinforce the same need for diagnostic confirmation^34,35,36^. Another limitation of the method is the risk of false-positive results in individuals with protein intake greater than 25 g within 4 hours before the test, as well as in those using protein supplements. Nevertheless, the study excluded these individuals to minimize this risk.

The substantial regional variability observed in the frequency of reduced kidney function (detected via POCTcr) across the federative units and BSN state chapters highlights significant disparities in the sociodemographic and clinical profiles of the evaluated populations. The increased proportion of self-declared Brown and Black individuals in certain regions, alongside the elevated prevalence of traditional risk factors such as arterial hypertension (HTN) and diabetes mellitus (DM), suggests underlying structural inequalities. These disparities potentially compromise both access to diagnosis and the quality of renal health care, underscoring the necessity for integrated and more effective prevention strategies within the primary care setting. It must be noted that the local screening actions were implemented in highly distinct settings—ranging from public squares to shopping centers aimed at populations with higher purchasing power—as defined by convenience. This variability inherent to the convenience sample methodology likely introduced selection bias, which may have influenced the sociodemographic and clinical characteristics of the resulting sample. These findings suggest that CKD in Brazil should be understood not merely as a clinical condition, but also as a marker of social vulnerability, necessitating public policies that rigorously address regional disparities and the social determinants of health.

The study indicated that approximately two-thirds of the participants were unaware of the risk factors for CKD (64.8%, based on the finding that only 35.2% reported knowledge of these factors) and, consequently, were not aware of their likelihood of developing kidney disease (Table 2).

The literature consistently demonstrates that knowledge concerning CKD and its risk factors remains limited across different contexts. In the United States, NHANES analyses indicate that only about 25% of adults with CKD stages G3–5 are aware of their diagnosis, while data stratified by KDIGO stages suggest awareness levels ranging between 9.6% and 49%, generally increasing with disease severity^37,38^. In low- and middle-income countries, the multicenter ISN-KDDC study, involving 12 countries, reported extremely low awareness (6–10%) among those affected by the disease^25^. In Brazil, data on awareness of CKD or its risk factors are scarce. National surveys such as VIGITEL and population-based studies confirm the high prevalence of HTN and DM, which are recognized CKD risk factors. This underscores the critically relevant finding of low kidney risk awareness from the present study, which contributes to the underdiagnosis of CKD, especially in its initial stages^28^. Population studies further demonstrate that a large proportion of individuals with proteinuria or reduced eGFR are unaware of their condition, with over 80% of individuals with proteinuria not reporting prior knowledge of the finding^39^. Furthermore, analyses of laboratory databases reveal the suboptimal utilization of recommended tests, such as the uACR, even in people already subjected to serum creatinine measurement, and most requests for kidney function assessment are performed by non-nephrologists, including in advanced stages of the disease^4^.

These findings point to a critical gap between the prevalence and recognition of CKD in the country, reinforcing the need to expand population- and professional-oriented educational strategies, strengthen screening within primary health care, and ensure greater adherence to guidelines for early detection and risk stratification. Accordingly, the EPI-CKD Brazil study presents important strengths and limitations that should be considered when interpreting its results. Among its main strengths is the large sample size, comprising more than 8,000 individuals from 20 of the 27 Brazilian federal units who had risk factors for CKD, which provides epidemiological robustness and national relevance. The study was conducted in the context of the World Kidney Day 2025, the largest global kidney health awareness campaign, whose primary objective is to increase public knowledge about kidney diseases. In Brazil, the campaign is organized by the Brazilian Society of Nephrology (BSN) and, in 2025, mobilized more than 1,200 activities nationwide.

The use of the Nova MaxPro^®^Creat/eGFR point-of-care testing (POCTcr) device in community settings enabled real-time estimation of the glomerular filtration rate without the need for a central laboratory, demonstrating practical feasibility for CKD screening in resource-limited environments. This unprecedented nationwide initiative generated original data on the frequency of reduced kidney function among at-risk populations and reinforces the relevance of incorporating screening strategies into primary care programs. In addition, the study contributed to increasing the visibility of CKD and highlighted the low level of public awareness regarding the disease, a critical aspect for informing public policies focused on prevention and kidney health care.

On the other hand, some limitations must be acknowledged. This is not a population-based study on the prevalence of CKD but rather a report of a screening campaign in which we identified participants with reduced eGFR who were considered potential cases of CKD. Considering this aspect, the characteristics of the participants may not be representative of the population that would be screened for CKD in care settings such as primary health care, medical offices, and emergency rooms. Therefore, we consider that, despite the significant sample size, our results cannot be extrapolated to all clinical care contexts involving patients with CKD risk factors. The cross-sectional design itself does not allow for the diagnosis of CKD nor for monitoring changes in participants’ eGFR over time. The analytical limitations inherent in the use of the POCTcr Nova MaxPro^®^Creat/eGFR, such as variability, bias in relation to standardized laboratory methods, and risk of error in values close to clinical cutoff points, require diagnostic confirmation in a laboratory before any clinical decision. Furthermore, the absence of albuminuria assessment limited the ability to identify possible cases of early CKD without reduced eGFR, since albuminuria alone may indicate CKD, although it also must be repeated for diagnostic confirmation. Finally, the results should be contextualized within the structural difficulties of the Brazilian health system, including barriers to accessing laboratory tests and nephrology specialists, which contribute to the underdiagnosis and undertreatment of CKD. Taken together, these aspects reinforce that, although the study demonstrates the feasibility of using rapid tests on a large scale as a screening strategy, it presents some methodological and analytical limitations, including the need for laboratory confirmation of eGFR by serum creatinine examination and longitudinal studies to confirm the diagnosis of CKD and guide effective public health interventions.

This study represents a crucial first step toward reducing the remaining significant gaps in care in the early stages of CKD, when timely interventions have the greatest potential to delay progression and reduce long-term morbidity and mortality^27,40^. These challenges become even more complex given regional and local disparities, often exacerbated by sociodemographic determinants such as race, ethnicity, health literacy, and barriers to access to the healthcare system—factors that influence both the risk of developing kidney disease and the possibility of receiving quality care^41,42^. In this context, strategies that expand access to easily applicable screening and diagnostic methods, including bedside testing, coupled with strengthening health education, improving primary care capacity, and formulating public policies that ensure equity, can promote early detection, appropriate management, and prevention of CKD at the population level^7,11,43^.

## Conclusion

The EPI-CKD Brazil study demonstrates the feasibility of using rapid creatinine tests on a large scale as a screening strategy for individuals at risk for CKD in Brazil, including at the primary health care level. Although it presents methodological limitations, our findings highlight the high frequency of reduced eGFR in at-risk populations, along with geographical variations, and the low population awareness of the risk factors that may lead to the disease. These results reinforce the urgency of implementing public policies that expand access to diagnostic methods, prioritize primary care, and strengthen health education. Timely interventions at this early stage have the potential to reduce disease progression, prevent complications, and decrease long-term morbidity and mortality, contributing to greater equity in kidney health care in the country.

## Data Availability

Data may be available upon request from the corresponding author as needed.
