# Supplementary material for: EPI-CKD Brazil: point-of-care screening for chronic kidney disease during World Kidney Day
Source: J Bras Nefrol. 2026 Apr 27;48(3):e20250298. doi: 10.1590/2175-8239-JBN-2025-0298en (PMC13169432; doi:10.1590/2175-8239-JBN-2025-0298en)
Supplement: Translation

# Translation: EPI-DRC Brasil: triagem da doença renal crônica com teste rápido de creatinina durante o Dia Mundial do Rim

**DOI:** 10.1590/2175-8239-JBN-2025-0298pt

**Authors:** Viviane Calice-Silva, Patrícia Abreu, Maria Eliete Pinheiros, Ana Katarina de Cerqueira Delgado Lopes, Karla Cristina Petruccelli, Ana Wanda Barreto Marinho, Ana Flávia Moura, Felipe Costa Neves, Cláudia Maria Costa de Oliveira, Silvana Daher Costa, Luiz Roberto de Sousa Ulisses, Fábio Humberto Ribeiro Paes Ferraz, Ramiele Aparecida Cruz Souza, Rachel Gatti Armani, Ciro Bruno Costa, Marcelo Garcia Tavares, Dyego José de Araújo Brito, Erika Cristina Ribeiro de Lima Carneiro, Luiz Gonzaga, Jenaine Oliveira Paixão, Helady Sanders-Pinheiro, Luis Claudio Santos Pinto, Lucas Acatauassu Nunes, Pablo Rodrigues Costa Alves, Amanda Maíra Damasceno Silva, René Scalet dos Santos, Suzana Ricardo Greffin, Gisele Vajgel, João Marcelo Medeiros de Andrade, Ginivaldo Victor Ribeiro do Nascimento, Igor Denizarde Bacelar Marques, Maria Izabel de Holanda, Pedro Tulio Monteiro de Castro Abreu Rocha, Kalyanne Cabral de Paula do O, Lelyanne Rodrigues Pereira Torauato, Cristina Karohl, Juliana Alves Manhaes de Andrade, Ariane Karen de Sousa, Denise Rodrigues Simão, Ana Carolina Nakamura Tome, Daniela Ponce, Fernanda Salomão Gorayeb-Polacchini, Maria Almerinda Vieira Fernandes Ribeiro Alves, Rubens Escobar Pires Lodi, Cinthia Montenegro, José A. Moura-Neto

**Affiliations:** 1 Sociedade Brasileira de Nefrologia, São Paulo, SP, Brasil.; 2 Universidade da Região de Joinville, Escola de Medicina, Joinville, SC, Brasil.; 3 Fundação Pró-Rim, Departamento de Pesquisa Científica, Joinville, SC, Brasil.; 4 Universidade Federal de São Paulo, São Paulo, SP, Brasil.; 5 Universidade Federal de Alagoas, Faculdade de Medicina, Maceió, AL, Brasil.; 6 Universidade Federal do Amazonas, Manaus, AM, Brasil.; 7 Escola Bahiana de Medicina e Saúde Pública, Salvador, BA, Brasil.; 8 Universidade Federal do Ceará, Hospital Geral de Fortaleza, Fortaleza, CE, Brasil.; 9 Nefroclínicas, Brasília, DF, Brasil.; 10 Universidade do Distrito Federal, Escola Superior de Ciências da Saúde, Brasília, DF, Brasil.; 11 Hospital Universitário Cassiano Antônio de Moraes, Vitória, ES, Brasil.; 12 Santa Casa de Misericórdia de Vitória, Escola Superior de Ciências, Vitória, ES, Brasil.; 13 Universidade Federal do Maranhão, Hospital Universitário, São Luís, MA, Brasil.; 14 Universidade Federal de Minas Gerais, Hospital das Clínicas, Belo Horizonte, MG, Brasil.; 15 Universidade Federal de Minas Gerais, Faculdade de Medicina, Belo Horizonte, MG, Brasil.; 16 Universidade Federal de Juiz de Fora, Faculdade de Medicina, Departamento de Clínica Médica, Juiz de Fora, MG, Brasil.; 17 Universidade Federal do Pará, Belém, PA, Brasil.; 18 Universidade Federal da Paraíba, Departamento de Medicina Interna, João Pessoa, PB, Brasil.; 19 Faculdades Pequeno Príncipe, Curitiba, PR, Brasil.; 20 Fundação Pró-Renal, Curitiba, PR, Brasil.; 21 Universidade Federal de Pernambuco, Recife, PE, Brasil.; 22 Instituto de Medicina Integral Prof. Fernando Figueira, Recife, PE, Brasil.; 23 Universidade Federal do Piauí, Teresina, PI, Brasil.; 24 Universidade Federal do Piauí, Hospital Universitário, Teresina, PI, Brasil.; 25 Hospital Federal de Bonsucesso, Rio de Janeiro, RJ, Brasil.; 26 Universidade Federal do Rio de Janeiro, Rio de Janeiro, RJ, Brasil.; 27 Rede Américas, Rio de Janeiro, RJ, Brasil.; 28 Hospital São Lucas Copacabana, Rio de Janeiro, RJ, Brasil.; 29 Universidade Federal do Rio Grande do Norte, Hospital Universitário Onofre Lopes, Natal, RN, Brasil.; 30 Universidade Federal de Ciências da Saúde de Porto Alegre, Porto Alegre, RS, Brasil.; 31 Santa Casa de Misericórdia de Porto Alegre, Serviço de Nefrologia, Porto Alegre, RS, Brasil.; 32 Universidade Federal do Rio Grande do Sul, Porto Alegre, RS, Brasil.; 33 Associação Renal Vida, Blumenau, SC, Brasil.; 34 Faculdade de Medicina de São José do Rio Preto, Hospital de Base, São José do Rio Preto, SP, Brasil.; 35 Universidade Estadual Paulista, Faculdade de Medicina de Botucatu, Botucatu, SP, Brasil.; 36 Universidade Estadual de Campinas, Faculdade de Ciências Médicas, Campinas, SP, Brasil.; 37 Nova Biomedical, Waltham, MA, USA.; 38 AstraZeneca Brasil, Cotia, SP, Brasil.

**Keywords:** Doença Renal Crônica, Prevalência, Fatores de Risco, Programas de Rastreamento, Taxa de Filtração Glomerular

## Abstract

**Introdução:**

A Doença Renal Crônica (DRC) é um problema de saúde pública, com prevalência estimada em 10% no Brasil, possivelmente subestimada por desigualdades regionais e limitações diagnósticas. O estudo EPI-DRC Brasil teve como objetivo estimar a prevalência de taxa de filtração glomerular estimada (TFGe) <60 mL/min/1,73m^2^ em adultos com fatores de risco, por meio de triagem com teste rápido de creatinina (POCTcr) durante o Dia Mundial do Rim/2025, além de avaliar variações regionais e preditores associados.

**Métodos:**

Estudo transversal multicêntrico, realizado em 20 regionais da Sociedade Brasileira de Nefrologia, incluindo indivíduos ≥18 anos com pelo menos um fator de risco para DRC (idade >60 anos, hipertensão (HA), diabetes (DM), doença cardiovascular, obesidade, uso crônico de anti-inflamatórios não esteroidais, histórico de injúria renal aguda, litíase renal bilateral ou história familiar de DRC). A função renal foi avaliada pelo teste rápido de creatinina (NovaMaxPro^®^-TFGe CKD-EPI 2021). Considerou-se TFGe reduzida quando <60 mL/min/1,73m^2^.

**Resultados:**

Analisamos 8.374 participantes (66,9% mulheres; mediana 58 anos; IMC 28,3kg/m^2^; 46,2% pardos). A frequência de TFGe reduzida foi 40,2% (n = 3.370), 36% estágios G3b–G5. Apenas 35,2% conheciam fatores de risco para DRC. Houve diferenças regionais significativas (de 27,9% [Bahia] a 55,2% [Paraná]; p < 0,001). Preditores independentes incluíram: idade (OR = 1,032; IC95%: 1,028–1,035), HA (OR = 1,27; IC95%: 1,15–1,40) e, como fator protetor, etnia indígena (OR = 0,40; IC95%: 0,20–0,89), p < 0,05. O risco aumentou 23% a cada fator de risco adicional.

**Conclusão:**

Observou-se elevada frequência de TFGe reduzida e baixo conhecimento sobre fatores de risco para DRC. O estudo demonstra a viabilidade do POCTcr em estratégias de rastreamento e reforça a necessidade de políticas públicas para ampliar o diagnóstico precoce e fortalecer a atenção primária.

## Introdução

A Doença Renal Crônica (DRC) é uma condição silenciosa, com prevalência global aproximada de 10%^1,2^. No Brasil, estudos observacionais indicam uma prevalência na população geral em torno de 8,9% a 11%^3,4,5^. Considerando o vasto território do país, as disparidades socioeconômicas e as variações no acesso aos serviços de saúde e aos exames diagnósticos, é provável que a prevalência real esteja subestimada no país^2^. Conhecer a epidemiologia da DRC é crucial para o desenvolvimento de planos estratégicos e políticas públicas voltadas à promoção da prevenção e ao diagnóstico precoce^2,6^. Essas estratégias são particularmente importantes em populações em risco, como pessoas com hipertensão arterial (HA), *diabetes mellitus* (DM), doenças cardiovasculares (DCV), obesidade, idade superior a 60 anos, histórico familiar de doenças renais, entre outros fatores^7^.

Dados recentes do Censo Brasileiro de Diálise de 2024 indicam uma crescente demanda por terapia renal substitutiva: em julho de 2024, haviam aproximadamente 172.585 pacientes em diálise crônica (prevalência de ~812 por milhão de habitantes), com 52.944 novos pacientes iniciando diálise naquele ano; notadamente, apenas ~5,6% foram tratados por diálise peritoneal^8^. Paralelamente, o modelo IMPACT CKD projeta que, de 2023 a 2032, o Brasil observará um crescimento substancial da DRC, especialmente nos estágios 3–5 (aumento de ~6,9%), com maior número de indivíduos necessitando de TRS, além de um incremento expressivo na morbidade, na mortalidade e nos custos^9^. Em conjunto, esses estudos ressaltam o imenso fardo clínico, humano e econômico da DRC para o sistema de saúde brasileiro. A crescente incidência e prevalência da DRC avançada superam a capacidade de tratamento, enquanto muitos casos em estágios iniciais permanecem não diagnosticados. Essa discrepância evidencia a necessidade urgente de fortalecer o rastreamento na atenção primária, ampliar o acesso ao diagnóstico (incluindo exames laboratoriais e testes rápidos) e implementar estratégias de prevenção voltadas a fatores de risco como diabetes, hipertensão e obesidade. Políticas públicas proativas e investimentos em detecção precoce podem mitigar a progressão para doença terminal, reduzir custos e aliviar a sobrecarga da infraestrutura de diálise em todo o país.

Atualmente, o diagnóstico da DRC baseia-se na estimativa da taxa de filtração glomerular (TFGe), calculada pela equação CKD-EPI (2021), a partir dos níveis séricos de creatinina, além da avaliação da presença de albuminúria por meio da relação albumina/creatinina em amostra isolada de urina (RACu). A presença de TFGe < 60 mL/min/1,73 m^2^ e/ou RACu > 30 mg/g, persistente por mais de 3 meses, associada ou não a alterações na morfologia renal, define a DRC. A classificação segue o sistema proposto pelo *Kidney Disease: Improving Global Outcomes (KDIGO)*, originalmente publicado em 2012 e atualizado em 2024^7^. Esse sistema combina TFGe e RACu para classificar o estágio da DRC, estratificar o risco de progressão da doença e orientar a necessidade de encaminhamento ao nefrologista. À medida que a DRC progride para estágios mais avançados, podem ocorrer complicações, culminando em falência renal e na necessidade de terapia renal substitutiva (TRS)^7^.

No entanto, um dos principais desafios para a detecção precoce é a dificuldade de realizar rastreamento em larga escala, já que a maioria dos exames requer infraestrutura laboratorial mínima para testes de creatinina e RACu. Nesse cenário, ferramentas aplicáveis à beira-leito ou em ações comunitárias, especialmente na atenção primária à saúde, podem contribuir para superar a ausência de triagem sistemática e favorecer o encaminhamento oportuno^10,11^. O Dia Mundial do Rim (DMR) é a maior campanha global de conscientização sobre saúde renal^12^. No Brasil, a Sociedade Brasileira de Nefrologia (SBN) coordena há mais de duas décadas ações de alcance nacional, envolvendo suas sociedades regionais em atividades de educação em saúde, rastreamento de fatores de risco e engajamento comunitário. Em 2025, a campanha atingiu todas as 20 regionais ativas da SBN, com foco em ampliar o conhecimento populacional sobre a DRC e integrar atividades científicas e de saúde pública e, pela primeira vez, incorporou nacionalmente ações de rastreamento com testes laboratoriais rápidos de creatinina.

Os testes diagnósticos rápidos (*point-of-care testing* [POCT]) têm se consolidado como ferramentas viáveis para avaliação da função renal fora do ambiente hospitalar^13^. O uso de dispositivos portáteis capazes de mensurar creatinina capilar e estimar a TFGe em tempo real possibilita rastrear indivíduos em campanhas públicas, ambulatórios de atenção primária, serviços de emergência e até mesmo em áreas remotas^13^. Apesar de variações em acurácia e precisão entre diferentes dispositivos, a experiência internacional indica que a adoção do POCT de creatinina (POCTcr) amplia a cobertura de rastreamento e contribui para reduzir o atraso no diagnóstico da DRC^14,15,16,17^.

Dessa forma, o estudo EPI-DRC Brasil teve como objetivo estimar a prevalência da TFGe < 60 mL/min/1,73 m^2^ em adultos com fatores de risco para DRC, por meio de triagem em amostra de conveniência utilizando POCTcr em todas as regiões do país durante as ações da Campanha do Dia Mundial do Rim de 2025, coordenada pela SBN e suas regionais. O estudo ainda teve como objetivo secundário avaliar as variações regionais da prevalência de TFGe < 60 mL/min/1,73 m^2^, identificar os principais fatores preditores associados e investigar o conhecimento da população sobre os fatores de risco para DRC.

## Métodos

### Desenho do Estudo

Trata-se de um estudo multicêntrico, transversal, com amostra de conveniência, realizado durante a campanha do DMR de 2025 no Brasil. Em razão de seu delineamento transversal e da utilização de uma única medida da TFGe por meio do POCTcr, o estudo não permite o diagnóstico definitivo de DRC, mas possibilita a identificação de reduções na TFGe potencialmente compatíveis com a doença, de modo que os participantes podem ser classificados apenas como tendo função renal reduzida ou como apresentando rastreamento positivo para essa condição, não sendo possível classificá-los como pacientes com DRC.

### Localização e Participantes

O estudo foi realizado como parte das ações da campanha do DMR de 2025 nas 20 regionais da SBN que compreendem 20 das 27 unidades federativas. O período de inclusão de participantes foi de 11/03/2025 a 30/04/2025. Por se tratar de um estudo realizado em uma campanha de conscientização sobre doenças renais, os indivíduos foram abordados e convidados a participar em locais públicos onde essas ações foram realizadas, o que pode ter levado a um potencial viés de seleção considerando os diferentes cenários nos quais a campanha foi realizada (ex.: shopping centers, praças públicas, entre outros). As cidades de recrutamento foram definidas pelas diretorias regionais da SBN que se inscreveram para participar do estudo.

Foram incluídos indivíduos adultos (≥ 18 anos) com pelo menos um fator de risco para DRC, descritos a seguir: 1) idade > 60 anos; 2) HA; 3) DM; 4) DCV; 5) obesidade (IMC ≥ 30 kg/m^2^); 6) histórico de injúria renal aguda (IRA); 7) histórico de litíase renal bilateral; 8) história de infecção urinária de repetição; 9) uso crônico de antiinflamatórios não esteroidais (AINEs); e 10) histórico familiar de DRC. Foram excluídos: ingestão > 25 g de proteína nas 4 horas que antecedem o teste de creatinina; uso de suplementos alimentares à base de proteína e/ou creatina de forma contínua; diagnóstico prévio de DRC de qualquer etiologia; estar em TRS (hemodiálise, hemodiafiltração, diálise peritoneal ou transplante renal); e participantes com dados incompletos na plataforma de inclusão de dados. Apenas indivíduos que concordaram em participar e assinaram o termo de consentimento livre e esclarecido (TCLE) foram incluídos. O estudo foi aprovado pelo Comitê de Ética em Pesquisa do Hospital Hans Dieter Schmidt (CAAE: 86100725.1.1001.5363).

### Procedimentos

Após assinatura do TCLE, cada participante respondeu a um questionário padronizado para coleta de dados demográficos, fatores de risco e comorbidades, além de uma pergunta sobre o seu conhecimento acerca dos fatores de risco para DRC (Pergunta: “Você sabe quais são os principais fatores de risco para desenvolver a doença renal crônica?” ( ) Sim ou ( ) Não). Em seguida, era realizado o POCTcr por punção digital para obtenção de amostra de sangue capilar, uma única vez, no medidor de creatinina Nova MaxPro^®^Creat/eGFR (Nova Biomedical, Waltham, MA, EUA), um analisador portátil com biossensor miniaturizado de uso único, projetado para testes de creatinina em sangue total. Pessoas com TFGe < 60 mL/min/1,73 m^2^ foram encaminhadas para posterior confirmação diagnóstica e avaliação com especialista, conforme o fluxo estabelecido por cada regional da SBN participante do estudo. Ao longo deste artigo, iremos nos referir a estes indivíduos como “rastreamento positivo para DRC”, para facilitar a compreensão dos dados. Essa denominação se justifica porque, pela metodologia empregada, esses participantes não preenchem os critérios diagnósticos laboratoriais de DRC, que incluem a avaliação da TFGe e/ou RACu em amostra isolada de urina em pelo menos dois momentos, com intervalo de 90 dias entre eles. Dessa forma, por se tratar de um estudo transversal, não podemos considerar o diagnóstico de DRC nessa população.

### Dosagem de Creatinina pelo Teste Rápido

O medidor de creatinina Nova MaxPro^®^Creat/eGFR (Nova Biomedical, Waltham, MA, EUA) permite avaliações simples, rápidas e precisas da função renal a partir de uma amostra de sangue capilar obtida por punção da polpa digital. Em estudos prévios, demonstrou uma sensibilidade de 98,9% e especificidade de 85,3% para detecção de doença renal, quando comparado ao método laboratorial padrão. O aparelho também realiza a estimativa da TFG pela equação CKD-EPI 2021^16^.

### Variáveis Coletadas

As variáveis demográficas, clínicas e de conhecimento sobre DRC foram coletadas e armazenadas, de forma desidentificada, pelo investigador principal, em plataforma eletrônica (REDCap), garantindo a privacidade dos participantes. Os dados demográficos e antropométricos coletados foram: idade, sexo, raça/etnia autorrelatada (branca, preta, parda [miscigenação entre brancos e negros], indígena e asiática), peso corporal (kg), altura (m), índice de massa corporal (IMC, kg/m^2^), prática de atividade física e tabagismo.

As comorbidades foram definidas como: diagnóstico prévio de HA (pressão arterial sistólica ≥ 140 mmHg ou diastólica ≥ 90 mmHg ou relato de uso de medicamento anti-hipertensivo); diagnóstico prévio de DM (relato do diagnóstico ou uso de medicamentos hipoglicemiantes); DCV (história de revascularização coronariana, infarto do miocárdio, acidente vascular cerebral [AVC] e/ou insuficiência cardíaca); obesidade (IMC ≥ 30 kg/m^2^); relato de uso crônico de AINEs (uso semanal); histórico de litíase renal bilateral (documentado por exame de imagem); infecção do trato urinário de repetição (sintomas compatíveis associados ao tratamento com antibióticos > 3 vezes/ano); e histórico prévio de IRA (piora temporária da função renal) e história familiar de DRC (familiares de primeiro ou segundo grau em tratamento conservador para DRC ou TRS).

### Análises Estatísticas

Análises descritivas foram realizadas para variáveis numéricas e apresentadas de acordo com a distribuição das variáveis. As variáveis categóricas foram apresentadas como frequências absolutas e relativas (n, %). Para a análise comparativa da prevalência por regional e das características da população com e sem rastreamento positivo para DRC (TFGe < 60 mL/min/1,73 m^2^), foram utilizados os testes qui-quadrado de amostras independentes e o teste de *Kruskal-Wallis*. A regressão logística foi utilizada para identificar fatores associados à DRC. O critério adotado para inclusão da variável na regressão logística foi p < 0,1 na análise univariada. O modelo de regressão logística não foi ajustado para agrupamento por região. A presença de colinearidade entre as variáveis independentes foi avaliada utilizando diagnóstico de colinearidade por meio de análise de regressão linear, com avaliação dos valores de *Variance Inflation Factor* (VIF) e *Tolerance*. Valores de VIF superiores a 5 e tolerância inferior a 0,2 foram considerados indicativos de colinearidade significativa. Também analisamos o risco adicional de rastreamento positivo para DRC de acordo com o número de fatores de risco, considerando os mais prevalentes no grupo estudado (HA, DM, idade > 60 anos, obesidade, e DCV). Os dados foram analisados no SPSS, versão 30.0 (IBM Corp., Armonk, NY, EUA).

## Resultados

No período do estudo, foram avaliados 8.428 indivíduos em 40 cidades de 20 unidades da federação (Figura 1). Destes, 8.374 participantes apresentaram dados completos e foram incluídos nas análises. A amostra foi composta predominantemente por mulheres (66,9%), com mediana de idade de 58 anos e IMC mediano de 28,3 kg/m^2^, e a maioria dos participantes se declarou parda (46,2%) (Tabela 1).

**Figura 1:** Distribuição dos participantes do estudo de acordo com as regionais da SBN e as unidades federativas do país.

**Tabela 1:** Dados demográficos, clínicos e de dosagem de creatinina da população incluída no estudo

Variável	N = 8374
Idade [anos, mediana (1º–3º quartis; mín.–máx.)]	58 (37–79) (18–103)
Sexo feminino (n, %)	5574 (66,9)
Raça	
Branco (n, %)	3078 (37,4)
Negro (n, %)	1294 (15,7)
Pardo (n, %)	3800 (46,2)
Indígena (n, %)	37 (0,4)
Asiático (n, %)	24 (0,3)
Tabagista (n, %)	1022 (12,3)
Sedentários (n, %)	4822 (58,2)
Idosos (n, %)	3994 (47,7)
HA (n, %)	4484 (54,1)
DM (n, %)	2358 (28,7)
DCV (n, %)	1200 (14,7)
Obesidade (n, %)	2598 (31,8)
IRA (n, %)	80 (1,2)
Litíase renal bilateral (n, %)	285 (4,4)
Uso crônico de AINEs (n, %)	312 (4,8)
História familiar DRC (n, %)	417 (6,4)
DM + HA (n, %)	1685 (20,1)
DM + HA + DCV (n, %)	418 (5)
DM + HA + DCV + Obesidade (n, %)	127 (1,5)
IMC [kg/m^2^, mediana (1º–3º quartis; mín.–máx.)]	28,3 (21,3–35,3) (13–64)
Creatinina [mg/dL, mediana (1º–3º quartis; mín.–máx.)]	1,1 (0,65–1,55) < 0,25 – > 7,0
TFGe [mL/min/1,73 m^2^, mediana (1º–3º quartis; mín.–máx.)]	65 (34 – > 90) (< 15 – > 90)
Faixas deTFGe (n, %)	
> 90 (n, %)	1441 (17,2)
60–89 (n, %)	3541 (42,3)
45–59 (n, %)	2180 (26,1)
30–44 (n, %)	968 (11,6)
15–29 (n, %)	185 (2,2)
< 15 (n, %)	50 (0,6)

Abreviaturas – HA: hipertensão arterial; DM: diabetes mellitus; DCV: doença cardiovascular; IRA: injúria renal aguda; AINEs: anti-inflamatórios não esteroidais; IMC: índice de massa corporal; TFGe: taxa de filtração glomerular estimada; DRC: doença renal crônica. IIQ: intervalo interquartil.

A frequência global de função renal reduzida (TFGe < 60 mL/min/1,73 m^2^) e, portanto, rastreamento positivo para DRC, foi de 40,2% (n = 3.370). Caso a DRC fosse confirmada, 36% destes indivíduos estariam nos estágios G3b– G5 (Tabela 1; Figura 2A e B; Figura 3). A comparação das características da população com e sem TFGe reduzida está apresentada na Tabela 2.

**Figura 2:** Distribuição dos participantes do estudo com TFGe < 60 mL/min/1,73 m^2^, de acordo com (A) regional da SBN e (B) unidades federativas do país.

**Figura 3:** Estágios da DRC na população estudada, caso a alteração na TFGe fosse confirmada.

Abreviaturas – G1 = ≥ 90 mL/min/1,73 m^2^; G2 = 60–89 mL/min/1,73 m^2^; G3a = 45–59 mL/min/1,73 m^2^; G3b = 30–44 mL/min/1,73 m^2^; G4 = 15–29 mL/min/1,73 m^2^; e G5 < 15 mL/min/1,73 m^2^.

**Tabela 2:** Comparação das características principais da população com e sem redução da função renal detectada pelo método aplicado

Variável	Todos N = 8374	TFGe ≥ 60 N = 5004	TFGe < 60 N = 3370	Valor P^*^
Idade [anos, mediana (1º–3º quartis; mín.–máx.)]	58 (37–79) (18–103)	55 (22) (18–96)	62 (19) (18–103)	< 0,001
Sexo feminino (n, %)	5574 (66,9)	3339 (67,2)	2235 (66,4)	0,43
Raça				< 0,001
Branco (n, %)	3078 (37,4)	1755 (35,7)	1323 (39,8)	
Negro (n, %)	1294 (15,7)	752 (15,3)	542 (16,3)	
Pardo (n, %)	3800 (46,2)	2363 (48,1)	1437 (43,2)	
Indígena (n, %)	37 (0,4)	28 (0,6)	9 (0,3)	
Asiático (n, %)	24 (0,3)	12 (0,2)	12 (0,4)	
Tabagista (n, %)	1022 (12,3)	611 (12,3)	411 (12,2)	0,91
Sedentário (n, %)	4822 (58,2)	2928 (59,3)	1894 (56,5)	0,012
Idosos (n, %)	3994 (47,7)	2017 (40,4)	1977 (58,4)	< 0,001
HA (n, %)	4484 (54,1)	2432 (49,3)	2052 (61,1)	< 0,001
DM (n, %)	2358 (28,7)	1374 (28,1)	984 (29,5)	0,16
DCV (n, %)	1200 (14,7)	636 (13,0)	564 (17,0)	< 0,001
Obesidade (n, %)	2598 (31,8)	1595 (32,7)	1003 (30,5)	0,03
Conhecimento fatores de risco (n, %)	2948 (35,2)	1851 (37,0)	1172 (34,8)	0,05
IMC [kg/m^2^, mediana (1º–3º quartis; mín.–máx.)]	28,3 (21,3–35,3) (13–64)	28,5 (21,5–35,5) (13–64)	28 (21–35) (15–56)	0,006
DM + HA (n, %)	80 (1,2)	914 (18,3)	771 (22,8)	< 0,001
DM + HA + DCV (n, %)	285 (4,4)	249 (4,4)	169 (5,8)	0,004
DM + HA + DCV + Obesidade (n, %)	312 (4,8)	72 (1,4%)	55 (1,6)	0,5
Creatinina [mg/dL, mediana (1º–3º quartis; mín.–máx.)]	1,1 (0,65–1,55) < 0,25 – > 7,0	0,94 (0,26) (< 0,25–1,63)	1,4 (0,4) (0,9 – > 7,0)	< 0,001
TFGe [mL/min/1,73 m^2^, mediana (1º–3º quartis; mín.–máx.)]	65 (34 – > 90) (< 15 – > 90)	78 (56 – > 90) (60 – > 90)	48 (35–59,5) (< 15 – 59,5)	< 0,001

Abreviaturas – HA: hipertensão arterial; DM: diabetes mellitus; DCV: doença cardiovascular; IMC: índice de massa corporal; TFGe: taxa de filtração glomerular estimada. IIQ: intervalo interquartil.

*Valores de P referentes aos testes de comparação entre os grupos DRC e não DRC. Para variáveis qualitativas, foi aplicado o teste qui-quadrado de amostras independentes; para variáveis quantitativas, o teste de *Kruskal-Wallis* para amostras independentes.

A frequência de função renal reduzida foi maior no Paraná (55,2%), no Piauí (53%), no Distrito Federal (51,3%) e em Minas Gerais (52,7%), e menor na Bahia (27,9%), no Amazonas (30,8%) e na Paraíba (30,7%), com diferença estatisticamente significativa entre as regionais (p < 0,001) (Figura 4). As características gerais da população estudada conforme cada regional da SBN variaram bastante e estão demonstradas na Tabela 3.

**Figura 4:** Prevalência de função renal reduzida (TFGe < 60 mL/min/1,73 m^2^) avaliada pelo teste rápido de creatinina, por regional da SBN.

**Tabela 3:** Comparação das características dos participantes entre regionais da SBN

Variável	Todos N = 8374	AL	AM	BA	CE	DF	ES	GO	MA	MT	MG	PA	PB	PR	PE	PI	RJ	RN	RS	SC	SP	Valor de p
Idade [anos, mediana (1º –3º quartis)]	58 (37–79)	52 (28–76)	54 (33–55)	59 (38–80)	50 (29–71)	53 (27–79)	50 (29–71)	56 (33–78)	53 (33–73)	50 (28–72)	63 (46–80)	53 (35–70)	62 (44–80)	67 (54–80)	61 (43–78)	62 (44–80)	57 (36–78)	69 (56–82)	61 (43–79)	54 (28–80)	63 (45–81)	< 0,001
Sexo feminino (n, %)	5574 (66,9)	170 (73,9)	259 (63,3)	337 (74,1)	360 (69,2)	323 (65,6)	296 (68,2)	242 (63,2)	294 (72,1)	291 (68,1)	295 (69,6)	233 (67,0)	341 (68,8)	120 (62,5)	246 (75,7)	280 (65,3)	307 (72,7)	283 (63,5)	185 (61,5)	279 (64,3)	433 (56,7)	< 0,001
Raça																						< 0,001
Branco (n, %)	3078 (37,4)	47 (22,5)	52 (13,0)	64 (14,1)	165 (32,3)	147 (29,5)	144 (33,2)	119 (30,8)	64 (15,7)	113 (26,5)	172 (41,3)	72 (20,7)	147 (29,8)	132 (76,3)	97 (30,6)	70 (17,2)	213 (51,7)	194 (43,9)	218 (72,2)	321 (74,1)	527 (69,3)	
Negro (n, %)	1294 (15,7)	42 (20,1)	30 (7,5)	203 (44,5)	39 (7,6)	76 (15,2)	70 (16,1)	37 (9,6)	106 (26,0)	75 (27,6)	100 (24,0)	34 (9,8)	78 (15,8)	10 (5,8)	55 (17,4)	76 (18,7)	82 (19,9)	52 (11,8)	39 (12,9)	33 (7,6)	57 (7,5)	
Pardo (n, %)	3800 (46,2)	119 (56,9)	310 (77,3)	188 (41,2)	306 (59,9)	269 (53,9)	218 (50,2)	229 (59,3)	236 (57,8)	234 (54,9)	142 (34,1)	241 (69,3)	264 (53,5)	29 (16,8)	164 (51,7)	259 (63,8)	117 (28,4)	196 (44,3)	43 (14,2)	78 (18,0)	158 (20,8)	
Indígena (n, %)	37 (0,4)	1 (0,5)	9 (2,2)	0	1 (0,2)	5 (1,0)	1 (0,2)	1 (0,3)	2 (0,5)	3 (0,7)	2 (0,5)	1 (0,3)	3 (0,6)	2 (1,2)	1 (0,3)	1 (0,2)	0	0	2 (0,7)	1 (0,2)	1 (0,1)	
Asiático (n, %)	24 (0,3)	0	0	1 (0,2)	0	2 (0,4)	1 (0,2)	0	0	1 (0,2)	0	0	1 (0,2)	0	0	0	0	0	0	0	18 (2,4)	
Tabagista (n, %)	1022 (12,3)	18 (7,9)	53 (13,1)	53 (11,7)	68 (13,1)	70 (13,8)	34 (7,8)	45 (11,7)	19 (4,7)	68 (15,9)	78 (18,5)	35 (10,1)	76 (15,3)	12 (6,6)	32 (9,9)	44 (10,6)	53 (12,6)	49 (11,0)	33 (10,9)	35 (8,1)	147 (19,2)	< 0,001
Sedentário (n, %)	4822 (58,2)	125 (56,1)	260 (64,0)	272 (59,8)	302 (58,1)	274 (54,3)	261 (60,4)	229 (59,3)	273 (67,2)	316 (74,4)	235 (56,4)	231 (66,4)	288 (58,4)	64 (35,4)	183 (57,7)	231 (51,7)	214 (50,8)	273 (61,1)	126 (41,9)	240 (55,4)	443 (58,2)	< 0,001
Idoso (n, %)	3994 (47,7)	83 (35,5)	157 (38,1)	225 (49,3)	244 (46,8)	153 (30,3)	165 (38,0)	150 (38,9)	129 (31,6)	116 (27,2)	268 (63,1)	109 (31,3)	295 (59,2)	153 (79,7)	178 (54,8)	256 (59,5)	195 (45,9)	244 (54,6)	241 (79,8)	172 (39,5)	461 (60,3)	< 0,001
HA (n, %)	4484 (54,1)	131 (67,2)	189 (46,6)	239 (52,4)	252 (48,5)	182 (36,1)	236 (54,5)	170 (44,9)	205 (50,4)	205 (48,0)	268 (63,2)	172 (49,4)	311 (62,7)	102 (54,8)	197 (60,8)	229 (54,3)	212 (50,1)	269 (60,6)	207 (68,5)	236 (54,4)	472 (61,9)	< 0,001
DM (n, %)	2358 (28,7)	57 (35,4)	100 (24,6)	155 (34,2)	140 (27,0)	84 (16,7)	115 (26,5)	76 (20,1)	103 (25,2)	86 (20,1)	157 (37,3)	90 (25,9)	163 (32,9)	56 (29,9)	108 (33,4)	102 (25,0)	106 (25,1)	163 (37,3)	99 (32,9)	110 (25,3)	288 (37,8)	< 0,001
DCV (n, %)	1200 (14,7)	34 (22,7)	57 (14,1)	63 (13,8)	75 (14,5)	57 (11,4)	28 (6,5)	44 (11,6)	73 (18,0)	56 (13,1)	81 (19,2)	32 (9,2)	89 (17,9)	35 (19,3)	48 (14,9)	59 (14,9)	39 (9,3)	87 (20,2)	58 (19,2)	53 (12,2)	145 (19,1)	< 0,001
Obesidade (n, %)	2598 (31,8)	122 (52,1)	149 (37,0)	111 (24,7)	194 (39,4)	138 (27,3)	152 (35,2)	129 (33,4)	125 (31,1)	166 (39,1)	122 (31,4)	109 (31,3)	182 (36,6)	53 (33,5)	91 (28,3)	112 (28,8)	142 (33,6)	90 (21,7)	18 (6,0)	164 (37,7)	229 (30,0)	< 0,001

Notas-Valores de P referentes aos testes de comparação entre os grupos DRC e não DRC. Para variáveis qualitativas, foi aplicado 0 teste qui-quadrado de amostras independentes; para variáveis quantitativas, 0 teste de *Kruskal-Wallis.*

Apenas 35,2% dos participantes declararam conhecer os fatores de risco para a DRC. Dentre os fatores de risco para DRC avaliados, os mais frequentes foram: HA, idade > 60 anos, obesidade e DM (Tabela 2). A distribuição do somatório dos fatores de risco está demonstrada na Tabela 2 e na Figura 5. A cada fator de risco adicional relatado, o risco de desenvolvimento de rastreamento positivo para DRC aumentou em 23% (OR = 1,23; IC95%: 1,20–1,30). Os principais preditores para TFGe reduzida foram idade (OR = 1,032; IC95%: 1,028–1,035), hipertensão arterial (OR = 1,27; IC95%: 1,15–1,4) e etnia indígena, que se mostrou fator protetor para TFGe reduzida (OR = 0,4; IC95%: 0,2–0,89), todos estatisticamente significativos (p < 0,05). Os demais resultados da regressão logística estão apresentados na Tabela 4. Não foi observada colinearidade significativa entre os fatores de risco incluídos no modelo.

**Figura 5:** Distribuição do somatório de fatores de risco mais prevalentes para DRC. Incluídos: hipertensão arterial, diabetes mellitus, idade > 60 anos, obesidade e doença cardiovascular.

**Tabela 4:** Preditores de taxa de filtração glomerular reduzida

Variable	Variável		Modelo 1		Modelo 2	
	OR (IC95%)	Valor de p	OR (IC95%)	Valor de p	OR (IC95%)	Valor de p
Idade	1,03 (1,02–1,04)	< 0,001	1,03 (1,02–1,04)	< 0,001	1,03 (1,02–1,04)	< 0,001
Sexo	1,04 (0,96–1,14)	0,43				
Etnia						
Branca	Referência		Referência		Referência	
Negra	0,96 (0,84–1,1)	0,5	1,07 (0,93–1,22)	0,34	1,03 (0,93–1,18)	0,7
Parda	0,81 (0,73–0,9)	< 0,001	0,94 (0,85–1,04)	0,2	0,94 (0,84–1,03)	0,15
Indígena	0,43 (0,22–0,91)	< 0,001	0,45 (0,21–0,98)	0,05	0,4 (0,2–0,9)	0,03
Asiática	1,33 (0,59–2,9)	0,5	1,07 (0,47–2,43)	0,8	1,2 (0,53–2,8)	0,6
Tabagista ativo	0,99 (0,87–1,13)	0,9				
Sedentário	0,89 (0,82–0,97)	0,01	0,97 (0,88–1,06)	0,5	0,95 (0,87–1,05)	0,3
HAS	1,61 (1,47–1,76)	< 0,001			1,22 (1,1–1,35)	< 0,001
DM	1,07 (0,97–1,2)	0,16				
DCV	1,37 (1,21–1,55)	< 0,001			1,09 (0,96–1,25)	0,2
Obesidade	0,9 (0,82–0,99)	0,03			1,09 (0,98–1,2)	0,1

Abreviaturas – HAS: hipertensão arterial sistêmica; DM: diabetes mellitus; DCV: doença cardiovascular.

Modelo 1: Ajustado para variáveis sociodemográficas que, na análise univariada, apresentaram p < 0,1.

Modelo 2: Modelo 01 + comorbidades mais prevalentes na população estudada.

## Discussão

O estudo EPI-DRC Brasil revelou elevada prevalência de função renal reduzida (TFGe < 60 mL/min/1,73 m^2^) entre indivíduos com fatores de risco avaliados por meio de teste rápido de creatinina e estimativa da TFG calculada pela equação CKD-EPI 2021, durante as ações da Campanha do Dia Mundial do Rim de 2025 nas 20 regionais da SBN. Devido ao desenho transversal do estudo, não é possível falar em prevalência de DRC, mas apenas em risco de DRC e em TFGe reduzida no momento da avaliação. Em relação aos dados de prevalência da doença, a literatura mundial demonstra números variáveis nas populações com e sem fatores de risco. Estimativas de países de média renda apontam prevalências de DRC na faixa de 9–17%, semelhantes à média global (11–13%)^18,19,20^. Levantamentos na China indicaram prevalência de 10,8% em 2009 e ~82 milhões de adultos com DRC em 2018–2019, sugerindo mudanças temporais possivelmente associadas a políticas públicas de incentivo ao diagnóstico precoce^21^. Na América Latina, dados do *Global Kidney Health Atlas* da ISN indicam prevalência média de ~10%, com ampla variabilidade entre países^2^. No Brasil, estudos observacionais indicam prevalência na população geral em torno de 8,9% a 11%^2,3^, além de subutilização da RACu no diagnóstico da DRC no Brasil^4^.

Estima-se que, em pacientes com fatores de risco para DRC, a prevalência global situa-se entre 20% e 30%, dependendo do perfil de risco específico e da região estudada^22,23,24^. Em um estudo do *International Society of Nephrology’s Kidney Disease Data Center* (ISN-KDDC), que incluiu 75.058 indivíduos de 12 países de baixa e média renda, a prevalência de DRC, analisada através da combinação de TFGe e RACu, foi de 14,3% (IC95%: 14,0–14,5) na população geral, e de 36,1% (IC95%: 34,7–37,6) em populações de alto risco (indivíduos com HA, DM ou DCV)^6,25^. Nesse estudo, as prevalências entre indivíduos de alto risco variaram de 19,4%, na Geórgia, no Leste Europeu, a 49,3% em Bangladesh, no Sul da Ásia^25^. Ainda, dados do programa KEEP (*Kidney Early Evaluation Program*), realizado nos EUA, demonstraram que a DRC é altamente prevalente entre indivíduos com fatores de risco conhecidos para a doença, com quase 30% apresentando sinais de lesão renal inicial (albuminúria) e 16% apresentando redução da função renal; destes, 14% correspondiam a diagnósticos novos de DRC^26^. No Brasil, dados de prevalência em pacientes com fatores de risco para DRC ainda são escassos.

Pela primeira vez, no Brasil, adultos com fatores de risco para DRC tiveram acesso ao rastreamento em larga escala por meio do POCTcr. Embora o rastreamento universal da população seja discutível em termos de custo-benefício, em grupos de risco, o uso combinado de TFGe e RACu é recomendado para a detecção precoce, permitindo o manejo otimizado da DRC com intervenções capazes de reduzir sua progressão^7^. Entretanto, as disparidades econômicas e as variações no acesso aos serviços de saúde e na capacidade diagnóstica têm um impacto profundo na epidemiologia, progressão e desfechos da doença renal em todo o mundo. Essas disparidades são evidentes tanto em países de baixa e média renda (*LMICs*) quanto em países de alta renda, mesmo naqueles com sistemas universais de saúde, como o Brasil^7,11,27^.

Considerando os fatores de risco para DRC analisados na população estudada, a maioria dos participantes era idosa (> 60 anos), hipertensa, diabética ou obesa, e cada fator de risco adicional presente aumentou o risco de rastreamento positivo para DRC em 23%. No Brasil, dados de inquéritos populacionais como o Sistema de Vigilância de Fatores de Risco e Proteção para Doenças Crônicas por Inquérito Telefônico (VIGITEL) e levantamentos demográficos do Instituto Brasileiro de Geografia e Estatística (IBGE) mostram a relevância dos principais fatores de risco para DRC na população idosa^28,29^. Atualmente, cerca de 15% da população brasileira tem 60 anos ou mais, faixa etária na qual a prevalência de HA ultrapassa 50% e a de DM aproxima-se de 20%, segundo estimativas do VIGITEL^28^. Além disso, aproximadamente um quarto dos brasileiros nessa faixa etária apresenta obesidade, refletindo o impacto do envelhecimento populacional associado a hábitos de vida que favorecem doenças crônicas^28,29^. Esses achados reforçam a carga crescente de condições cardiometabólicas e seu papel na determinação da prevalência e progressão da DRC no país.

A detecção precoce da DRC por meio de testes laboratoriais à beira-leito (POCT) torna-se uma peça-chave para identificar indivíduos sob risco. Esses testes têm como principal objetivo a avaliação rápida da função renal (creatinina capilar com cálculo de TFGe no próprio dispositivo) e a detecção de lesão renal por meio da avaliação da presença de albumina na urina. Na triagem e monitoramento da DRC, o POCTcr pode ser utilizado para estratificar pacientes quando a coleta venosa ou laboratórios centrais não estão prontamente disponíveis (*e.g.*, clínicas de extensão, setores de radiologia e prontossocorros), embora a acurácia e a precisão variem de acordo com o equipamento e o contexto clínico^30,31,32^.

Em nosso estudo, todos os participantes detectados com TFGe < 60mL/min/1,73 m^2^ foram referenciados para confirmação laboratorial com exame de creatinina sérica, e aqueles com resultados confirmando DRC foram encaminhados para avaliação médica conforme o estágio da doença, de acordo com os Protocolos Clínicos e Diretrizes Terapêuticas (PCDT) 2024^33^, e com fluxos organizados por cada regional. Esses dados, entretanto, não foram capturados pelo presente estudo, não sendo possível confirmar o diagnóstico de DRC com as informações obtidas. O teste rápido Nova MaxPro^®^Creat/eGFR apresenta taxas elevadas de sensibilidade (98%) e especificidade (85%), mas não descarta a possibilidade de resultados falsos positivos e indica a necessidade de confirmação posterior por teste padrão-ouro^16^. Dados da literatura sobre outros dispositivos de testagem da creatinina à beira-leito reforçam a mesma necessidade de posterior confirmação diagnóstica^34,35,36^. Outra limitação do método é o risco de falso positivo entre indivíduos com ingestão proteica superior a 25g nas 4 horas que antecedem o teste, bem como aqueles que utilizam suplementos proteicos. No entanto, o estudo excluiu estes indivíduos com o intuito de minimizar esse risco.

A grande variação encontrada na frequência da redução da função renal por meio do teste rápido entre unidades federativas e regionais da SBN evidencia diferenças relevantes no perfil sociodemográfico e clínico entre as regiões avaliadas. A maior proporção de indivíduos autodeclarados pardos e pretos em alguns estados, bem como a elevada frequência de fatores de risco tradicionais, como hipertensão arterial e diabetes mellitus, entre outros, podem demonstrar desigualdades estruturais que impactam tanto o acesso ao diagnóstico quanto a qualidade do cuidado em saúde renal e reforçam a necessidade de estratégias de prevenção integradas e mais eficazes no âmbito da atenção primária. Cabe destacar que as ações locais foram realizadas em espaços bastante distintos, desde praças públicas até centros comerciais voltados para um público de maior poder aquisitivo, definidos por conveniência, o que pode ter influenciado as características sociodemográficas e clínicas da amostra. Esses achados sugerem que a DRC no Brasil deve ser compreendida não apenas como uma condição clínica, mas também como um marcador de vulnerabilidade social, exigindo políticas públicas que considerem as disparidades regionais e os determinantes sociais da saúde.

O estudo apontou que cerca de 2/3 dos participantes desconheciam os fatores de risco para DRC e, consequentemente, não sabiam que estavam suscetíveis ao desenvolvimento de doença renal. A literatura demonstra de forma consistente que o conhecimento sobre a DRC e seus fatores de risco permanece limitado em diferentes contextos. Nos Estados Unidos, análises do NHANES indicam que apenas cerca de 25% dos adultos com DRC estágios G3–G5 têm conhecimento do diagnóstico, enquanto dados estratificados segundo estágios da DRC pelo KDIGO apontam níveis de conhecimento variando entre 9,6% e 49%, aumentando com a gravidade da doença^37,38^. Em países de baixa e média renda, o estudo multicêntrico ISN-KDDC, envolvendo 12 países, demonstrou nível de conhecimento extremamente baixo (6–10%) entre os afetados pela doença^25^. No Brasil, não há dados sobre o conhecimento da DRC ou de seus fatores de risco. No entanto, inquéritos nacionais, como o VIGITEL, bem como estudos de base populacional, confirmam alta prevalência de HA e DM, reconhecidos fatores de risco para DRC. Esse cenário torna particularmente relevante o achado deste estudo, que demonstrou baixo reconhecimento do risco renal, levando ao subdiagnóstico dessa condição, especialmente nos estágios iniciais^28^. Estudos populacionais evidenciam que grande parte dos portadores de proteinúria ou TFGe reduzida desconhece sua condição, com mais de 80% dos indivíduos com proteinúria não relatando conhecimento prévio do achado^39^. Além disso, análises de bases laboratoriais revelam subutilização de exames recomendados, como a RACu, mesmo em pessoas já submetidas à dosagem de creatinina sérica, e a maioria das solicitações de função renal é realizada por médicos não nefrologistas, inclusive em estágios avançados da doença^4^. Esses dados apontam para uma lacuna crítica entre prevalência e reconhecimento da DRC no país, reforçando a necessidade de ampliar estratégias de educação populacional e profissional, fortalecer o rastreamento na atenção primária e garantir maior adesão às diretrizes de detecção precoce e estratificação de risco.

Assim sendo, o estudo EPI-DRC Brasil apresenta importantes pontos fortes e limitações que devem ser considerados na interpretação dos resultados. Entre os principais méritos, destacase a ampla amostra estudada, com mais de 8.000 indivíduos de 20 das 27 unidades federativas do país, com fatores de risco para DRC, conferindo robustez e relevância epidemiológica. O estudo foi realizado no contexto do DMR 2025, a maior campanha global de saúde renal, cujo principal objetivo é aumentar o conhecimento da população sobre as doenças renais, organizada no Brasil pela SBN e que, em 2025, mobilizou mais de 1.200 atividades em todo o país. A utilização do POCTcr Nova MaxPro^®^Creat/eGFR em ambiente comunitário permitiu a mensuração em tempo real, sem necessidade de laboratório central, da estimativa da TFG, demonstrando viabilidade prática para rastreamento da DRC em cenários com recursos limitados. Essa iniciativa, inédita em escala nacional, gerou dados originais sobre a prevalência de função renal reduzida em populações de risco e reforça a pertinência de incorporar estratégias de triagem em programas de atenção primária. Além disso, o estudo contribuiu para aumentar a visibilidade da DRC e para evidenciar o baixo nível de conhecimento da população sobre a doença, aspecto fundamental para orientar políticas públicas de educação voltadas à prevenção e ao cuidado renal.

Por outro lado, algumas limitações devem ser reconhecidas. Este não é um estudo populacional sobre prevalência da DRC, mas sim um relatório de uma campanha de rastreio, na qual identificamos participantes com TFGe reduzida, que foram considerados potenciais indivíduos com DRC. Considerando este aspecto, as características dos participantes podem não ser representativas da população que seria rastreada para DRC em cenários de assistência, como atenção primária à saúde, consultórios médicos e pronto-atendimentos. Dessa forma, consideramos que, apesar do tamanho amostral expressivo, nossos resultados não podem ser extrapolados para todo e qualquer contexto de assistência ao paciente com fatores de risco para DRC. O próprio delineamento transversal não permite a realização do diagnóstico da doença, nem o acompanhamento da progressão da TFGe dos participantes ao longo do tempo. As restrições analíticas inerentes ao uso do POCTcr Nova MaxPro^®^Creat/eGFR, como variabilidade, viés em relação a métodos laboratoriais padronizados e risco de erro em valores próximos aos pontos de corte clínicos, exigem confirmação diagnóstica em laboratório antes de qualquer decisão clínica. Ademais, a ausência de avaliação de albuminúria limitou a capacidade de identificar possíveis casos de DRC inicial sem redução da TFGe, uma vez que essa variável, quando avaliada isoladamente, também pode indicar a presença de doença renal, embora esses achados também precisem ser repetidos para confirmação diagnóstica. Finalmente, os resultados devem ser contextualizados frente às dificuldades estruturais do sistema de saúde brasileiro, incluindo barreiras de acesso a exames laboratoriais e a especialistas em nefrologia, que contribuem para o subdiagnóstico e o subtratamento da DRC. Em conjunto, esses aspectos reforçam que, embora o estudo demonstre a viabilidade do uso de testes rápidos em larga escala como estratégia de triagem, existem limitações metodológicas e analíticas, como a necessidade de confirmação laboratorial da TFGe por exame de creatinina sérica e de estudos longitudinais para confirmar o diagnóstico de DRC e orientar intervenções efetivas em saúde pública.

Este estudo representa um primeiro passo fundamental para reduzir as lacunas assistenciais ainda marcantes nos estágios iniciais da DRC, momento em que intervenções oportunas têm maior potencial de retardar a progressão e reduzir a morbimortalidade a longo prazo^27,40^. Tais desafios tornam-se ainda mais complexos diante das disparidades regionais e locais, frequentemente agravadas por determinantes sociodemográficos como raça, etnia, letramento em saúde e barreiras de acesso ao sistema, fatores que influenciam tanto o risco de desenvolver doença renal quanto a possibilidade de receber cuidados de qualidade^41,42^. Nesse cenário, estratégias que ampliem o acesso a métodos de rastreamento e diagnóstico de fácil aplicabilidade, incluindo testes à beira-leito, associadas ao fortalecimento da educação em saúde, à qualificação da atenção primária e à formulação de políticas públicas que garantam equidade, podem favorecer a detecção precoce, o manejo adequado e a prevenção da DRC em escala populacional^7,11,43^.

## Conclusão

O estudo EPI-DRC Brasil demonstra a viabilidade do uso de testes rápidos de creatinina em larga escala como estratégia de rastreamento de pessoas em risco para DRC no Brasil, inclusive no âmbito da atenção primária à saúde. Embora apresente limitações metodológicas, nossos achados evidenciam a elevada frequência de TFGe reduzida em populações em risco, bem como suas variações geográficas, além da baixa consciência da população sobre quais fatores de risco podem levar ao desenvolvimento da doença. Esses resultados reforçam a urgência de implementar políticas públicas que ampliem o acesso a métodos diagnósticos, priorizem a atenção primária e fortaleçam a educação em saúde. Intervenções oportunas nesses estágios iniciais têm potencial de reduzir a progressão da doença, prevenir complicações e diminuir a morbimortalidade a longo prazo, contribuindo para maior equidade no cuidado da saúde renal no país.

## Data Availability

Os dados poderão ser disponibilizados mediante solicitação ao autor correspondente, conforme necessidade.
